# Multi-Parameter Optimization and Imaging Performance Evaluation of a 16 × 1 Ce:GAGG Linear Scintillator Detector: A Monte Carlo Simulation Study

**DOI:** 10.3390/ma19214516

**Published:** 2026-10-27

**Authors:** Zhu Liu, Yanggang Jia, Zhaoran Guan, Xinya Wang, Meng Cao, Haofei Huang, Linjun Wang, Jian Huang

**Affiliations:** 1 State Key Laboratory of Materials for Advanced Nuclear Energy & School of Materials Science and Engineering, Shanghai University, Shanghai 200444, Chinamonicazubek@163.com (Z.G.);; 2 Zhejiang Institute of Advanced Materials, Shanghai University, Jiaxing 314113, China; 3 Shanghai Engineering Research Center for Integrated Circuits and Advanced Display Materials, Shanghai University, Shanghai 200444, China

**Keywords:** Ce:GAGG, linear scintillator detector, Geant4, sub-pixel scanning, optical crosstalk, spatial resolution

## Abstract

Ce:GAGG linear scintillator detectors are promising for high-resolution radiation imaging, but signal sharing near pixel boundaries can affect position resolution. A Geant4 model of a 16 × 1 array was used to assess the lateral pixel dimension (PT), reflector thickness (REF), reflector material, metal interlayer and anti-scatter grid (ASG). The Gaussian-equivalent full width at half maximum (FWHM) of the reconstructed-position distribution and the spatial frequency at 50% modulation transfer (MTF50) were evaluated at central and boundary irradiation positions. PT = 1.0 mm, REF = 0.3 mm and MgO gave a favorable balance between central and boundary responses; the metal interlayer had little influence. A W ASG with 0.60 mm bars and a height of 5.0 mm was selected by comparing total and neighboring-channel energy-deposition tallies in a scattering-phantom geometry. Relative to the reference configuration, the phase-integrated adjacent-channel optical-response fraction decreased from 13.66% to 1.53%, the slanted-edge 10–90% width decreased from 2.951 to 1.876 mm, and the imaging MTF50 point estimate increased from 0.163 to 0.256 cycles mm^−1^. The integrated recorded optical-photon count decreased by 47.2%. In an ASG-free optical comparison, bar-pattern modulation increased from 0.361 to 0.668. The numerical results apply to the specified simulation and scoring conditions.

## 1. Introduction

Ce:GAGG (Ce-doped Gd_3_Al_2_Ga_3_O_12_) has attracted sustained attention in nuclear medical imaging, γ/X-ray detection, and high-resolution indirect detectors because of its high light yield, high density, fast decay time, non-hygroscopic nature, and good spectral matching with silicon-based photodetectors [1,2,3]. In recent years, with the continuous development of silicon-based photodetectors such as SiPMs in high-fill-factor arrays, miniaturized packaging, and high-gain, low-noise readout, compact detection modules based on GAGG scintillators coupled with silicon photodetectors have shown strong application potential in PET, space payloads, and medical X-ray detection [4,5,6,7,8]. Previous studies have shown that the compositional design of GAGG, crystal size, readout scheme, and application scenario can all significantly affect its energy resolution, light output, and imaging performance [2,6,9,10].

For pixelated scintillator arrays, spatial response depends on pixel geometry, optical boundary conditions, reflector properties, and the readout configuration, in addition to scintillator light yield [11,12,13,14]. Monte Carlo optical-transport simulations can describe the redistribution of scintillation light among readout channels [12,13,14,15,16,17]. With irradiation near the center of a pixel, the response is generally dominated by that pixel. Near an inter-pixel boundary, the detected signal may be shared between adjacent channels, changing the position-response distribution and the accuracy of position reconstruction. The present study, therefore, evaluates the response over different irradiation positions within a pixel pitch rather than using central irradiation alone. Central or phase-averaged metrics may not reveal degraded performance near the pixel boundary [18,19,20]. The full width at half maximum (FWHM) is used here as a Gaussian-equivalent measure of the reconstructed-position spread; Gaussian-equivalent MTF50 is the spatial frequency at which the corresponding normalized modulation transfer function decreases to 0.5. These quantities are derived from the simulated position response and do not represent independent experimental measurements.

On this basis, a Geant4 optical-transport model was established for a 16 × 1 Ce:GAGG linear scintillator detector. The optimization comprised two complementary stages: PT, REF, reflector material, and metal interlayer were first screened to control internal scintillation-light transport and pixel-boundary sharing, after which the ASG was evaluated as an external scatter-control component. A center–avg–worst framework was used to compare central response, full-phase stability, and the most unfavorable boundary response. The selected parameter combination was then assessed using phase–pixel response maps, slanted-edge analysis, and pattern-level imaging.

## 2. Detector Geometry Model and Simulation Method

### 2.1. Geometry Model and Material Settings

**Scheme 1:**
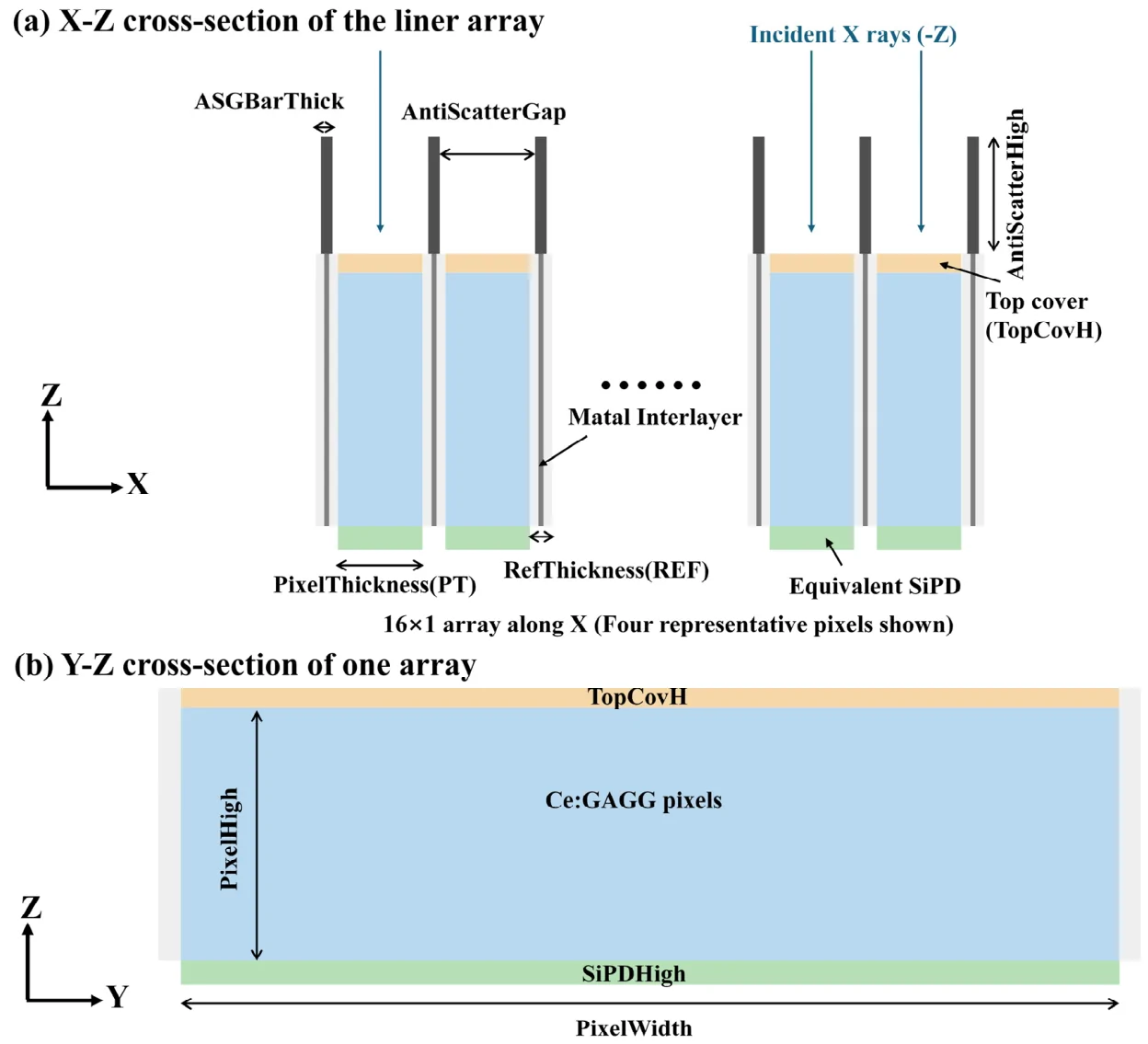
Detector geometry used in the Geant4 model: (**a**) X–Z cross-section of the 16 × 1 array showing PT, REF, the metal interlayer, top cover, equivalent SiPD layer, ASG bar thickness, ASG height, ASG placement interval, coordinate system, and incident direction; (**b**) Y–Z cross-section of one pixel showing PixelWidth, PixelHigh, TopCovH, and SiPDHigh.

The detector geometry is illustrated in Scheme 1. The model comprised 16 Ce:GAGG pixels arranged along X, 1 pixel along Y, and an incident/thickness direction along Z. The X-direction pixel dimension was denoted PT (Geant4 command: PixelThickness) and was scanned at 0.6, 0.8, 1.0, and 1.2 mm. PixelWidth and PixelHigh were fixed at 16 and 8 mm, respectively. The inter-pixel reflector thickness was denoted REF (RefThickness) and was scanned at 0.3, 0.5, and 0.8 mm.

In the X × Y × Z dimension convention, PixelThickness (PT) denotes the lateral dimension of each Ce:GAGG element along the 16-pixel array direction (X), PixelWidth denotes its length along Y (16 mm), and PixelHigh denotes its depth along the incident radiation direction (Z, 8 mm). Thus, PT is a Geant4 input-variable name and must not be interpreted as the 8 mm X-ray absorption depth. The reflector thickness REF is measured along X between adjacent scintillator elements; the corresponding center-to-center pitch is p = PT + REF.

An equivalent silicon photodetector (SiPD) layer with SiPDHigh = 0.075 mm was placed on the readout side. A 0.05 mm top-cover layer (TopCovH) was placed on the incident side. In each configuration, the top cover used the same material and UNIFIED optical-surface parameters as the selected reflector; no independent bulk absorption or transmission coefficient was assigned to this layer. The reflector and top-cover optical parameters are listed in Table S1. The ASG was located above the top cover. Its bar thickness, height, and placement interval corresponded to the Geant4 parameters ASGBarThick, AntiScatterHigh, and AntiScatterGap, respectively; a placement interval of 1 denotes one pixel pitch.

The reflector materials were TiO_2_, BaSO_4_, MgO, and ESR, and the candidate metal interlayers were W, Pb, Cu, and Al with thicknesses of 0, 0.05, 0.10, and 0.15 mm. Ce:GAGG was assigned a density of 6.62 g cm^−3^, a refractive index of 1.9 within the emission band, an effective optical absorption length of 300 mm, a scintillation yield of 46,000 photons MeV^−1^, and a decay time of 90 ns. The long absorption length was used consistently in all cases to isolate the relative effects of the structural parameters.

The Ce:GAGG refractive index n = 1.9 and optical absorption length of 300 mm are effective, wavelength-independent inputs adopted for controlled structural comparisons; they were not measured for a specific crystal batch in this study. The 300 mm value represents a weak-bulk-absorption approximation over the modeled 8 mm detector depth, rather than an experimentally established attenuation length. Real dispersion in refractive index, absorption length, scintillation emission, and interface response can change Fresnel reflection, total internal reflection, optical-photon survival, and, hence, the measured channel weights. Accordingly, the numerical improvements and reflector ranking are conditional on the optical properties in Table S1, and their robustness to wavelength-dependent Ce:GAGG properties has not been established.

The center-to-center pixel pitch *p* was defined as the sum of the lateral pixel dimension PT and the inter-pixel reflector thickness REF:

(1) p=PT+REF

where *p* defines the basic scale for sub-pixel scan-step normalization, boundary-position determination, and one-dimensional reconstruction coordinates.

### 2.2. Physical Processes and Definition of Sub-Pixel Scanning

All Monte Carlo simulations were performed using Geant4 version 11.2.2 (geant4-11-02-patch-02). The physics configuration comprised the FTFP_BERT reference physics list, G4EmStandardPhysics_option4 for electromagnetic interactions, G4OpticalPhysics for scintillation and optical-photon transport, and the decay and radioactive-decay physics constructors. A production cut of 0.1 mm was applied. Cherenkov emission, optical Rayleigh scattering, and Mie scattering were disabled; scintillation secondaries were tracked first. X/γ-ray interactions, secondary-electron transport, scintillation, optical reflection and refraction, and photon absorption were considered within the defined material and surface models. For the position-response calculations, a narrow monoenergetic photon beam was translated along the array direction (X), and the normalized irradiation coordinate was defined as:

(2) $$x_{in}=x_{c}+phase\cdot p$$

where *x_c_* is the reference-pixel center and *p* is the pixel pitch. Irradiation phase *ϕ* is dimensionless, with *ϕ* = 0 at the pixel center and *ϕ* ≈ ±0.5 near the adjacent pitch boundaries.

Source and acquisition parameters were specified for each calculation (Table S2). The PT sub-pixel scans employed 60 keV photons and a 0.04 × 16 mm^2^ irradiation strip, with 200,000 primary photons per sampled position in the documented PT series. The image-response calculations used 80 keV photons, with 500 primary photons at each of 61 strip positions and 1000 primary photons at each of 21 slanted-edge positions, respectively. For the ASG assessment at a bar thickness of 0.60 mm and height of 10 mm, 20,000 primary 80 keV photons were generated from a 16 × 16 mm^2^ planar source at z = −150 mm. A 100 mm high-density polyethylene (HDPE) scatterer and a 5 mm scatterer-to-detector separation were used. The source dimensions, simulated event numbers and scattering configurations are summarized in Table S2.

The terms center, avg and worst refer to the response at *ϕ* = 0, the mean response over the sampled phases, and the least favorable response within the boundary-sensitive phase interval, respectively. These definitions are applied consistently to the position-resolution and adjacent-channel response metrics.

### 2.3. Position Reconstruction, Evaluation Metrics, and Optimization Strategy

Event position was reconstructed using the standard centroid method. If the center coordinate of the *i*-th pixel is *x*_i_ and its corresponding effective response is *S*_i_, then the reconstructed position *x_rec_* is given by:

(3) $$x_{rec}=\frac{\sum_{i}S_{i}x_{i}}{\sum_{i}S_{i}}$$

where *S_i_* is the optical-photon count recorded at the SiPD interface of channel *i*. To characterize the signal sharing between the primary pixel and its neighboring pixels, the normalized response fraction was further defined as:

(4) $$w_{i}=\frac{S_{i}}{\sum_{j}S_{j}}$$

At a fixed irradiation position, the event-by-event reconstructed coordinates form a position-response distribution. Its standard deviation *σ* was obtained from the distribution and converted to Gaussian-equivalent measures of spatial spread and frequency response. The associated Gaussian representation is:

(5) $$LSF(x)=A\cdot \exp\left[-\frac{{\left(x-x_{0}\right)}^{2}}{2\sigma^{2}}\right]+B$$

where *A* is the amplitude, *x*_0_ is the distribution center, *σ* is the standard deviation and *B* is a constant background. Here, *σ* was calculated directly from the reconstructed-position distribution and was not obtained by fitting the distribution to a single Gaussian function. The corresponding Gaussian-equivalent full width at half maximum (FWHM) is:

(6) $$FWHM=2\sqrt{2\ln2}\sigma$$

If the Gaussian LSF is Fourier transformed, the modulation transfer function (MTF) can be expressed as:

(7) $$MTF(f)=\frac{\left|\int_{-\infty }^{+\infty }LSF(x)e^{-i2\pi fx}dx\right|}{\left|\int_{-\infty }^{+\infty }LSF(x)dx\right|}$$

The Gaussian-equivalent MTF50, defined as the spatial frequency at which the normalized Gaussian modulation transfer function decreases to 0.5, is therefore:

(8) $$MTF50=\frac{\sqrt{\ln2}}{\sqrt{2}\pi \sigma }$$

Response distributions near pixel boundaries may be asymmetric or multimodal owing to position-dependent light sharing and changes in the dominant readout channel. Accordingly, a Gaussian-equivalent FWHM or MTF50 is a measure of distribution width under the Gaussian approximation, rather than a unique description of the actual boundary-response shape. The phase-resolved response and slanted-edge analysis are, therefore, considered alongside these scalar measures when comparing detector configurations.

For the 0.60 mm W-ASG-bar and 10 mm ASG-height configuration, statistical uncertainty in the neighboring-channel Screen-step fraction was estimated by bootstrap resampling of primary-photon histories. Each resample comprised 20,000 histories drawn with replacement, including histories without a Screen record; 1000 resamples were used. The standard error and the 95% percentile confidence interval were obtained from the bootstrap distribution.

The neighboring-to-dominant-channel response ratio was defined using the recorded optical responses *S*_i_ of the 16 readout channels:

(9) $$ctk_{ratio}=\left(S_{m-1}+S_{m+1}\right)/S_{m}$$

where *m* = arg ma*x*_i_ *S*_i_ denotes the channel with the highest response at the corresponding irradiation position. The numerator is the sum of the responses in its existing immediately adjacent channels. Because the denominator is *S*_m_ rather than the sum over all channels, this quantity is a neighboring-to-dominant response ratio, not a fraction of the total recorded light. For the ASG study, the positive-energy-deposition steps recorded in the Screen scoring tree were used to define the total tally Tot and the neighboring-channel tally *Nei*_abs_ (CopyNumber = 7 or 9), with the corresponding fraction:

(10) $$Nei_{frac}=\frac{Nei_{abs}}{Tot}$$

A neighboring-to-dominant response ratio approaching unity at *ϕ* ≈ ±0.5 indicates comparable signal amplitudes in the nearest-neighbor channels and the dominant channel. It cannot be interpreted as a probability of photons crossing the inter-pixel reflector because both irradiation-position-dependent energy deposition and optical transport contribute to channel sharing. A measurement of physical optical leakage would require photon-origin and collection-channel information.

The sub-pixel neighboring-to-dominant response ratio and the phase-integrated adjacent-channel fraction *CTK*_int_ (Section 2.4) have different denominators. Neither is equivalent to a photon-origin-tagged cross-pixel transfer probability. Likewise, the ASG quantities Tot and Neiabs are energy-deposition-step tallies, and Neifrac = Neiabs/Tot is their neighboring-channel fraction; these quantities do not distinguish unscattered primary radiation from scattered radiation.

To avoid overly optimistic conclusions based only on central or averaged values, the evaluation results for each parameter set were classified into three categories: center, avg, and worst. For any evaluation quantity *Q*(phase), the average metric was defined as:

(11) $$Q_{avg}=\frac{1}{N}\sum_{k=1}^{N}Q(phase_{k})$$

The worst-case metric was defined as the maximum FWHM, the minimum MTF50, or the maximum crosstalk within the boundary-sensitive region, that is:

(12) $$FWHM_{worst}=\max_{\phi \in \Omega_{edge}}FWHM(\phi )$$

(13) $$MTF50_{worst}=\min_{\phi \in \Omega_{edge}}MTF50(\phi )$$

(14) $$ctk_{worst}=\max_{\phi \in \Omega_{edge}}ctk(\phi )$$

The parameters were assessed sequentially rather than by simultaneous global optimization. PT, REF, reflector material, and metal interlayer were examined in that order for the optical position response. The selected structure was then used in the ASG thickness and height scans under the scattering-phantom condition. At each stage, the preceding parameter choices were retained while the next parameter was varied. No weighted objective function was applied. The worst-case metric constrained boundary response, avg described variation across the sampled phases, and center assessed the response at central irradiation. FWHM and MTF50 were considered together because both were derived from the same reconstructed-position distribution; the neighboring-channel response ratio provided an additional measure of signal sharing. For the ASG, the neighboring-channel step tally and total step tally were compared jointly. Candidate settings were evaluated by their observed trade-offs over the discrete parameter ranges. The selected settings are, therefore, conditional choices within the investigated configurations, not the result of global optimization.

### 2.4. Image Reconstruction and Imaging Evaluation Methods

For image-level evaluation, phase–pixel response maps, slanted-edge analysis, Siemens-star imaging, and one-dimensional bar-pattern targets were used. For a phase–pixel response map, the normalized responses of all pixels at different sub-pixel positions were arranged into a two-dimensional matrix:

(15) $$R(m,\phi )=\frac{S_{m}(\phi )}{\sum_{n}S_{n}(\phi )}$$

Here, *m* denotes the pixel index. The phase–pixel map displays the main response band, boundary transition and lateral tails.

The slanted-edge target produces a spatial transition between irradiated and non-irradiated regions. The edge spread function (ESF) is the response as a function of position normal to this boundary, and its spatial derivative defines the line spread function (LSF):

(16) $$LSF(x)=\frac{dESF(x)}{dx}$$

The imaging modulation transfer function (MTF) was obtained by Fourier transforming the LSF and normalizing the result:

(17) $$MTF(f)=\frac{\left|F[LSF(x)]\right|}{|F[LSF(x)]{|}_{f=0}}$$

For the one-dimensional bar pattern, modulation was calculated from the mean responses of the bright- and dark-bar pixel groups:

(18) $$M=\frac{{\bar{I}}_{\mathrm{bright}}-{\bar{I}}_{\mathrm{dark}}}{{\bar{I}}_{\mathrm{bright}}+{\bar{I}}_{\mathrm{dark}}}$$

According to the geometrical overlap between each pixel active area and the open bars of the ideal target, pixels with an overlap fraction greater than 50% were assigned to the bright-bar group, whereas those with an overlap fraction below 50% were assigned to the dark-bar group. Edge pixels whose active areas were not fully contained within the lateral extent of the bar-pattern source were excluded. This definition reduces the influence of local statistical fluctuations associated with a single maximum or minimum value and provides a more stable measure of the overall separation between bright and dark bars.

For image-level comparison, the phase-integrated adjacent-channel response fraction was evaluated over the full scan. Unlike the position-dependent neighboring response, this integrated quantity describes the aggregate response. At each scan step *k*, the pixel with the greatest optical response was *m_k_* = arg max*_j_S_j_*(*ϕ_k_*). The phase-integrated fraction *CTK*_int_ was defined as:

(19) $$CTK_{\mathrm{int}}=\frac{\sum_{k=1}^{N}\left[S_{m_{k}-1}(\phi_{k})+S_{m_{k}+1}(\phi_{k})\right]}{\sum_{k=1}^{N}\sum_{j}S_{j}(\phi_{k})}$$

When the dominant-response pixel was located at an array edge, only the existing adjacent pixel was included. The denominator also represents the total recorded optical-photon count over the complete phase scan and was used to evaluate the response loss accompanying crosstalk suppression.

For the phase-scan analysis, uncertainty was estimated from 2000 bootstrap resamples of complete primary histories, including histories with zero recorded optical response. EventID indices were resampled jointly across scan positions within each configuration, whereas the baseline and optimized configurations were resampled independently. CTKint and the total optical-response count were recalculated for each replicate. The slanted-edge fit-stability analysis is summarized in Supplementary Information, Tables S4 and S5, and Figure S6.

For the slanted-edge images, the responses from the central seven scan rows were first averaged, and the physical pixel-center coordinates were calculated using the actual pixel pitch of each configuration. The rising part of the ESF within the common illuminated interval from *x* = −2.0 to 7.2 mm was then fitted using an error function. The terminal decrease caused by the finite source extent and detector boundary was excluded from the fit. The fitting function was:

(20) $$ESF(x)=B+\frac{A}{2}\left[1+\mathrm{erf}\left(\frac{x-x_{0}}{\sqrt{2}\sigma_{e}}\right)\right]$$

The corresponding 10–90% edge-spread width and imaging MTF50 were calculated as:

(21) $$W_{10-90}=2\sqrt{2}{\mathrm{erf}}^{-1}(0.8)\sigma_{e}$$

(22) $$MTF50_{\mathrm{edge}}=\frac{\sqrt{\ln2}}{\sqrt{2}\pi \sigma_{e}}$$

Because only 16 discrete pixels were available along the X direction, direct Fourier transformation of the truncated discrete LSF was susceptible to sampling-range and boundary effects. Therefore, the discrete LSF was retained to visualize changes in the response gradient, whereas the quantitative imaging MTF50 values were calculated from the Gaussian LSF corresponding to the error-function fit.

### 2.5. Code Verification and Method Consistency Assessment

The Geant4 optical model and post-processing code were checked against published LSF and MTF results, as shown in Figure S1 [21]. The simulated LSF reproduces the central peak and approximately symmetric long-tail distribution, and the MTF follows the reported frequency-dependent decay. The simulated MTF0.2 frequency is approximately 1258 lp mm^−1^ compared with 1254 lp mm^−1^ in the published result, a relative deviation of about 0.3%. This comparison supports the consistency of the simulation and post-processing workflow for relative spatial-response analysis; it is code and method verification rather than direct experimental validation of the detector geometry.

## 3. Results and Discussion

### 3.1. Effect of Pixel Size

**Figure 1:**
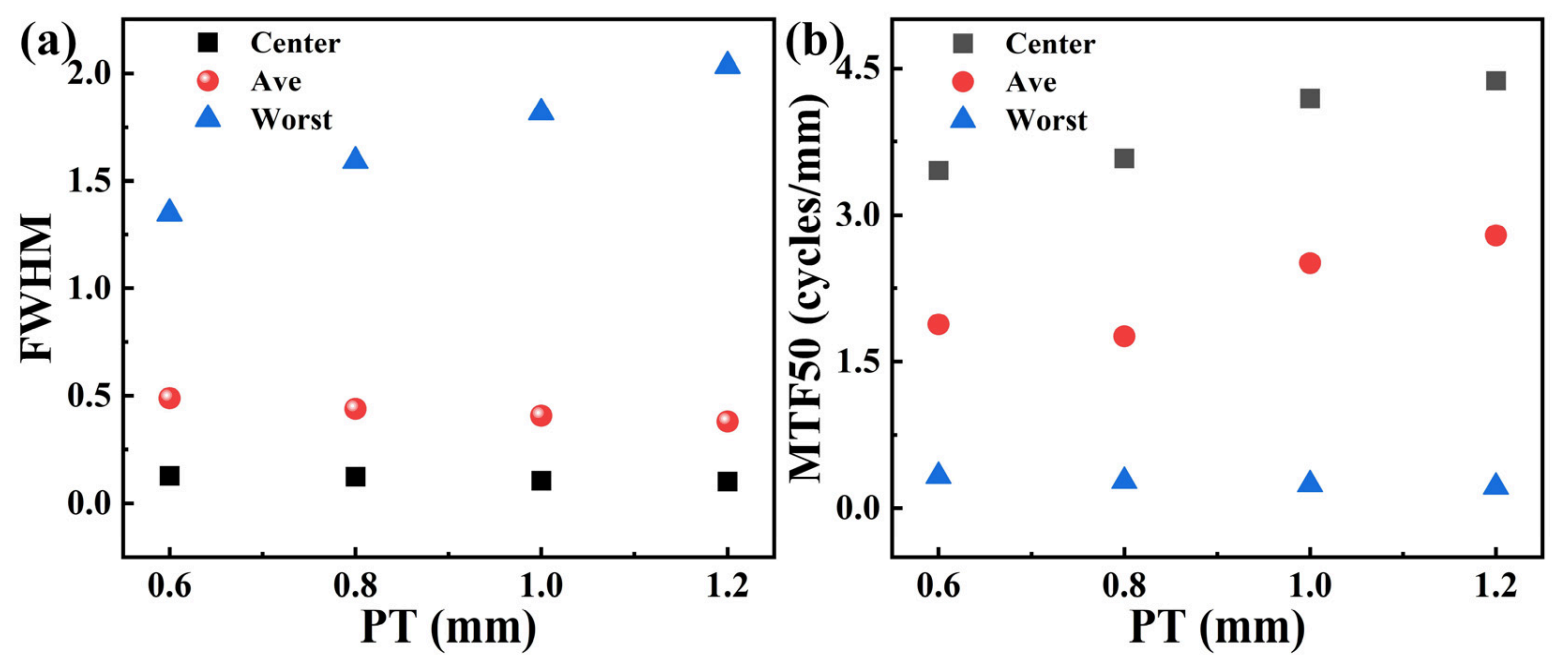
Effect of PT (0.6–1.2 mm) on the spatial-resolution performance with REF = 0.5 mm and TiO_2_ fixed: (**a**) FWHM; (**b**) MTF50. Center denotes phase = 0, avg denotes the full-phase mean, and worst denotes the most unfavorable boundary value.

Figure 1 shows the variation in the spatial-resolution performance of the 16 × 1 linear detector with different pixel sizes (PT), where center, avg, and worst represent the central-incidence condition, the full-phase average condition, and the most unfavorable boundary condition, respectively. As PT increases from 0.6 to 1.2 mm, the spatial-resolution performance under central incidence improves continuously. Specifically, FWHM_center_ decreases from 0.1277 to 0.1009 mm, while MTF50_center_ increases from 3.4566 to 4.3742 cycles/mm. This trend indicates that, near the pixel center, a larger lateral pixel size strengthens the dominant collection of scintillation light by the primary pixel and, thus, improves the stability of local readout for central events. Physically, scintillation photons generated under central incidence are collected mainly within the primary pixel, while the fraction escaping laterally into neighboring pixels remains relatively low. As a result, the detector response is governed primarily by photon statistical fluctuations within the primary pixel, limited lateral spreading, and the light-collection efficiency at the readout-side coupling interface [22,23,24]. Under this transport condition, which is dominated by single-pixel collection, a larger PT more effectively suppresses sidewall competition. This helps reduce main-peak broadening caused by lateral light sharing and preserves more high-frequency information [25,26]. In contrast to the continuous improvement in the center metrics, the FWHM under the worst condition increases with PT, while MTF50 decreases further. This behavior suggests that a larger pixel size does not simultaneously improve the positioning performance near pixel boundaries. The key reason is that when the incident position approaches the pixel boundary, i.e., phase ≈ ±0.5, the detector response is no longer dominated by a single pixel. Instead, it enters a shared-readout regime in which adjacent pixels contribute simultaneously. In this case, position reconstruction depends not on the absolute signal collected by one pixel but on the local sensitivity of the response difference between the left and right pixels to position changes. When this sensitivity decreases and boundary-response nonlinearity becomes stronger, the same level of photon statistical fluctuation is mapped into a larger positional uncertainty [22,27,28]. Therefore, increasing PT improves the single-pixel constraint for central events, but it does not simultaneously enhance the response stability within the boundary-sharing region. This is the fundamental reason for the clear divergence between the center and worst metrics [29].

Figure S2 provides more direct evidence of this mechanism in the phase dimension. For all PT values, the high-MTF50/low-FWHM region remains concentrated around phase ≈ 0, whereas the low-MTF50/high-FWHM band is consistently locked near phase ≈ ±0.5. This shows that the worst-case condition is not a random result dominated by statistical noise but a deterministic consequence of the pixel boundary as a geometrically and optically sensitive region. In particular, when PT = 1.2 mm, the performance degradation at the boundary is already large enough to offset the gain obtained at the center. This result indicates that optimization based only on central response or averaged metrics can easily lead to an overly optimistic assessment of structural improvement [27,29].

### 3.2. Optimization of Reflector Thickness

**Figure 2:**
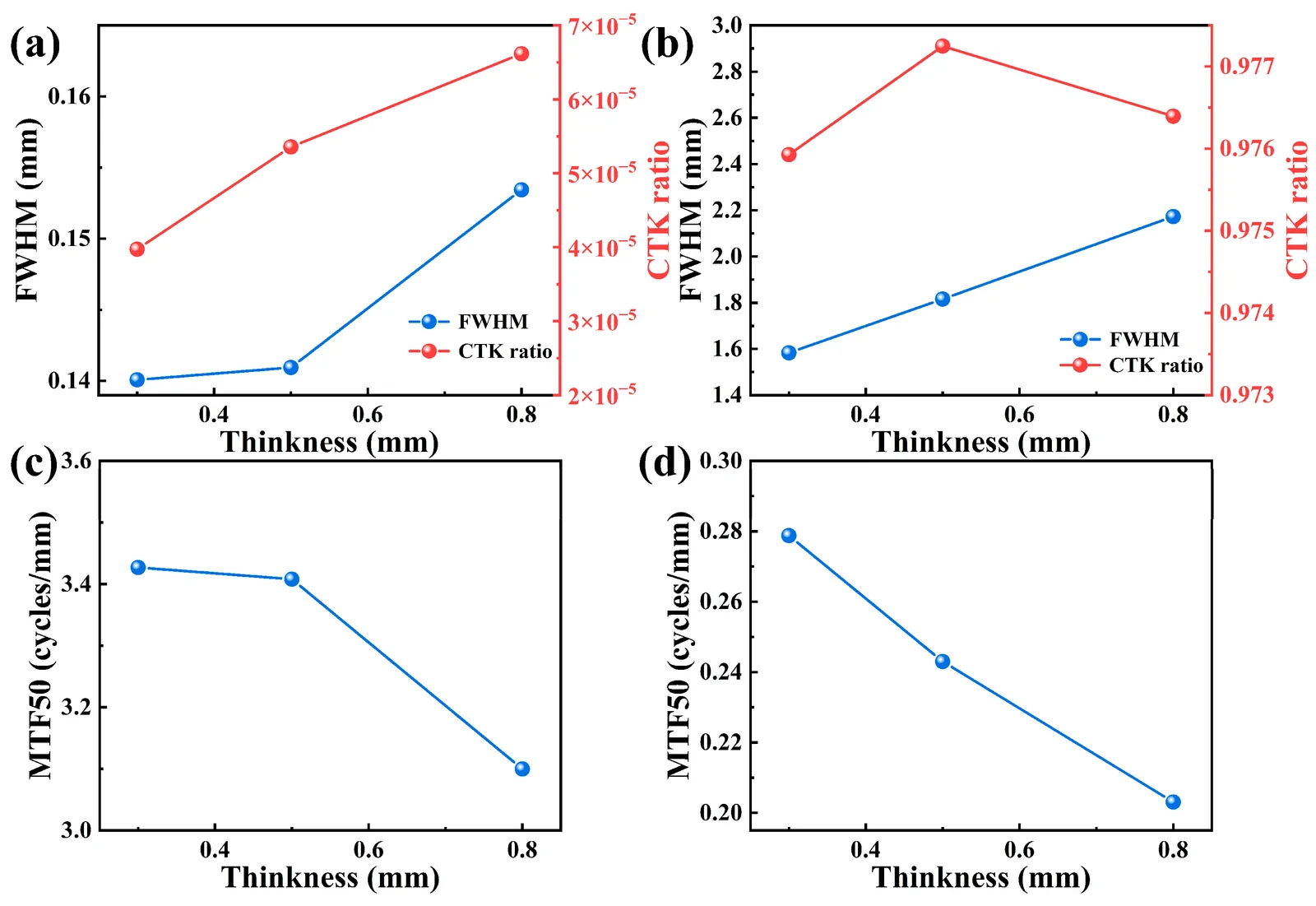
Effect of REF (0.3, 0.5, and 0.8 mm) at PT = 1.0 mm with TiO_2_ fixed: (**a**) FWHM and neighboring-to-dominant-channel response ratio (ctk_ratio_) at phase = 0; (**b**) FWHM and neighboring-to-dominant-channel response ratio (ctk_ratio_) at phase = ±0.5; (**c**) MTF50 at phase = 0; (**d**) MTF50 at phase = ±0.5.

The REF sweep was performed at PT = 1.0 mm with TiO_2_ fixed as the reflector material; the other structural parameters were unchanged. Thus, the thickness trend discussed in this section applies to the TiO_2_ boundary model used for the sweep, while material-dependent effects are evaluated separately in Section 3.3. Figure 2 compares REF = 0.3, 0.5, and 0.8 mm. Reflector thickness changes the effective pixel pitch and the lateral transport path near the pixel boundary, producing a much stronger effect under boundary incidence than under central incidence.

As shown in Figure 2, the influence of reflector thickness differs clearly between the central and boundary conditions. Under central incidence, when REF increases from 0.3 to 0.8 mm, FWHM shows only a slight increase, while MTF50 exhibits only a moderate decrease at the largest thickness. This indicates that, within the range considered in this study, reflector thickness is not the dominant factor controlling the central-positioning performance. The reason is that scintillation photons generated by central events are mainly transported within the primary pixel and collected by the bottom readout side. Their transport is dominated by near-axial propagation and a limited number of reflections, while the fraction leaking laterally into neighboring pixels remains low. As a result, the detector response is governed mainly by photon statistical fluctuations within the primary pixel, limited lateral spreading, and the light-collection efficiency at the crystal–SiPD coupling interface [13,22,25]. In contrast, the boundary condition is much more sensitive to changes in REF. As REF increases from 0.3 to 0.8 mm, the FWHM under boundary incidence increases continuously, while MTF50 decreases markedly. A plausible explanation is that both the high-frequency response and the position-retention capability near the boundary deteriorate as the reflector becomes thicker. The underlying reason is not simply the increase in geometric thickness itself but a stronger redistribution of the optical transport process for boundary events. On the one hand, the pixel pitch increases accordingly, so the same normalized phase corresponds to a larger actual lateral displacement, which further broadens the boundary transition region in real space. On the other hand, a thicker isolation layer changes the photon propagation geometry near the boundary, so that some photons must travel a longer lateral path and undergo more sidewall reflections before being collected by either the primary pixel or the neighboring pixel [17,25,26]. A longer propagation path and more reflections enhance the randomization of photon propagation directions and broaden the lateral distribution at the readout side, which ultimately reduces the boundary response gradient and lowers MTF50 [24,28].

Taken together, Figure 2a–d show that, within the parameter range studied here, a thinner reflector is more favorable for maintaining higher position discrimination in boundary regions while still providing basic optical isolation. This result indicates that, for linear scintillator detectors, reflector-thickness optimization should not focus only on increasing nominal isolation capability but should also pay close attention to its influence on the response gradient and spatial-frequency retention in the boundary-sharing region [25,26,27].

### 3.3. Effect of Reflector Material

**Figure 3:**
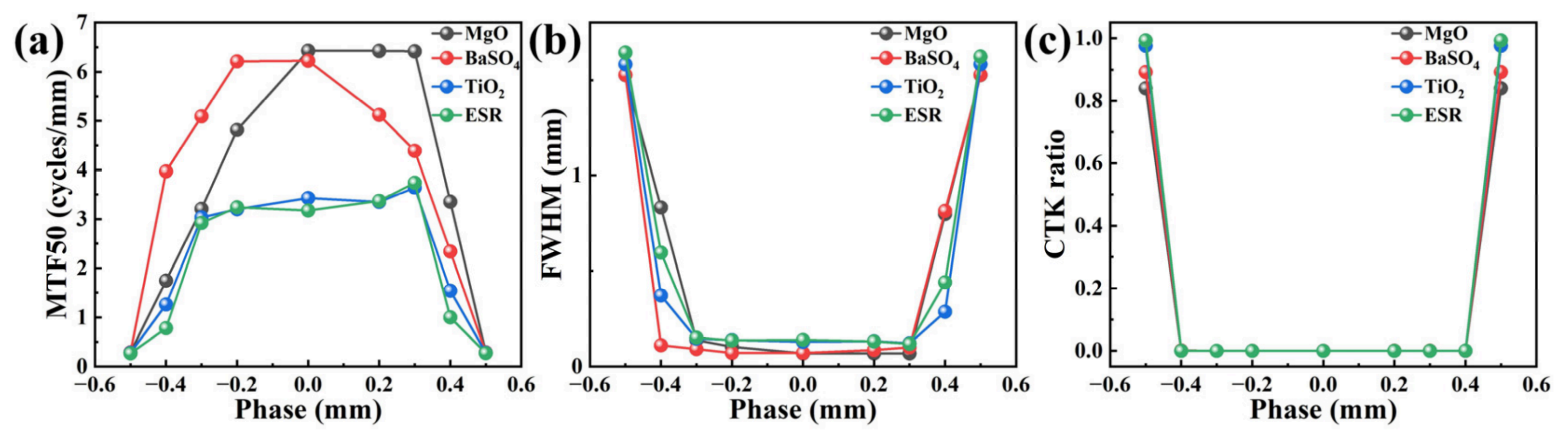
Phase-dependent response for TiO_2_, BaSO_4_, MgO, and ESR at PT = 1.0 mm and REF = 0.3 mm: (**a**) MTF50; (**b**) FWHM; (**c**) neighboring-to-dominant-channel response ratio (ctk_ratio_).

Figure 3 shows the full phase–response curves for different reflector materials under fixed conditions of PT = 1.0 mm and REF = 0.3 mm. The differences among these materials are reflected not only in the absolute values of the response metrics but also in the overall shape of the phase–response curves. For MTF50 and FWHM, the central region around phase ≈ 0 mainly reflects the local light-collection capability of the primary pixel, whereas the boundary region around phase ≈ ±0.5 corresponds to the most sensitive regime dominated by inter-pixel sharing. As a result, the influence of reflector material becomes much more pronounced near the boundary. MgO and BaSO_4_ show higher MTF50 and lower FWHM in the central region, whereas ESR and TiO_2_ exhibit flatter but overall weaker responses during phase scanning. Meanwhile, the *CTK* ratio rises rapidly mainly near the boundary phases, indicating that the effect of reflector material on boundary neighboring-channel response is much stronger than its effect under central incidence [13,17,25]. The underlying reason is that different reflector layers do not simply provide a binary boundary of reflective or non-reflective. Instead, they reshape the angular distribution and return paths of photons at the sidewalls through their respective reflection phase functions, surface roughness, and diffuse-to-specular reflection ratios [13,15,16]. For pixelated scintillators, an excessively strong specular component tends to preserve the original propagation direction of photons, allowing high-angle components to undergo long-range sliding along the sidewalls. In contrast, moderate diffuse scattering is more effective at breaking up large-angle propagation components, shortening the lateral transport correlation length, and promoting more localized light recovery within the primary pixel [17,25,26]. Therefore, the differences in the phase–response curves essentially reflect the ability of different reflector materials to regulate the lateral transport mode of scintillation photons, rather than merely differences in static reflectivity.

**Figure 4:**
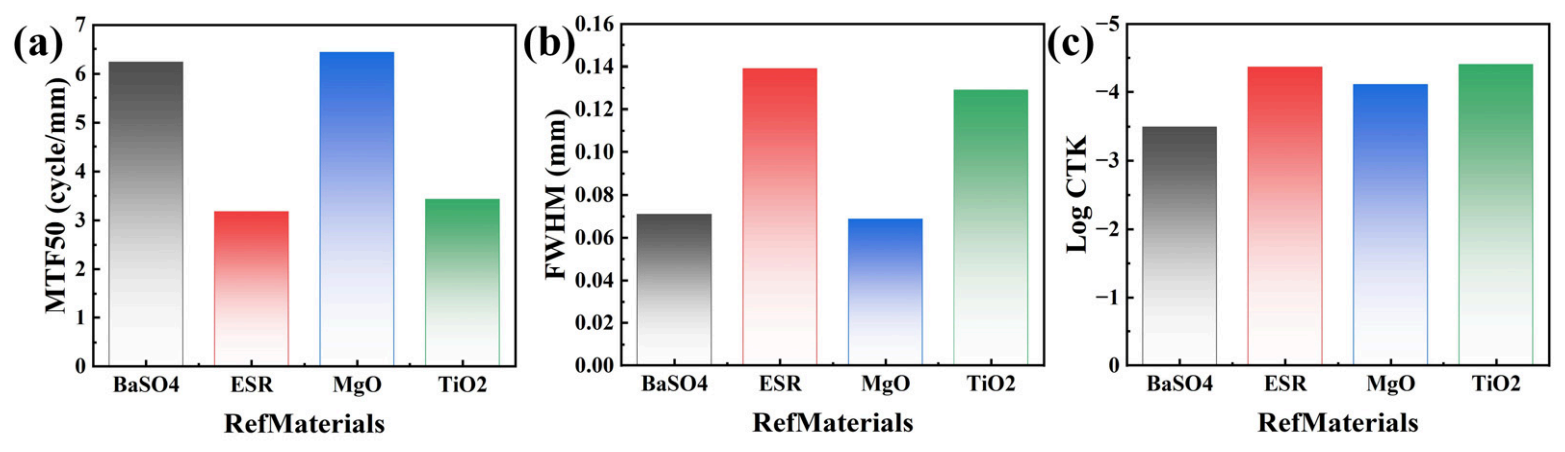
Response at phase = 0 for TiO_2_, BaSO_4_, MgO, and ESR with PT = 1.0 mm and REF = 0.3 mm: (**a**) MTF50; (**b**) FWHM; (**c**) log10 neighboring-to-dominant-channel response ratio (*CTK*_ratio_).

Figure 4 compares MTF50, FWHM, and log10 optical crosstalk for the four reflector materials at phase = 0. MgO gives the highest MTF50 and lowest FWHM, followed closely by BaSO_4_. In contrast, the neighboring-to-dominant-channel response ratios are all close to zero: 3.97 × 10^−5^ for TiO_2_, 4.27 × 10^−5^ for ESR, 7.66 × 10^−5^ for MgO, and 3.22 × 10^−4^ for BaSO_4_. Under centered incidence, the source and pixel geometry are symmetric, and the primary pixel dominates the response, so only a very small fraction reaches adjacent pixels. Reflector-dependent differences at phase = 0 are, therefore, expressed mainly through the return path and collection distribution within the primary pixel, which changes FWHM and MTF50 but has little effect on the neighboring-channel weights [13,22,25].

**Figure 5:**
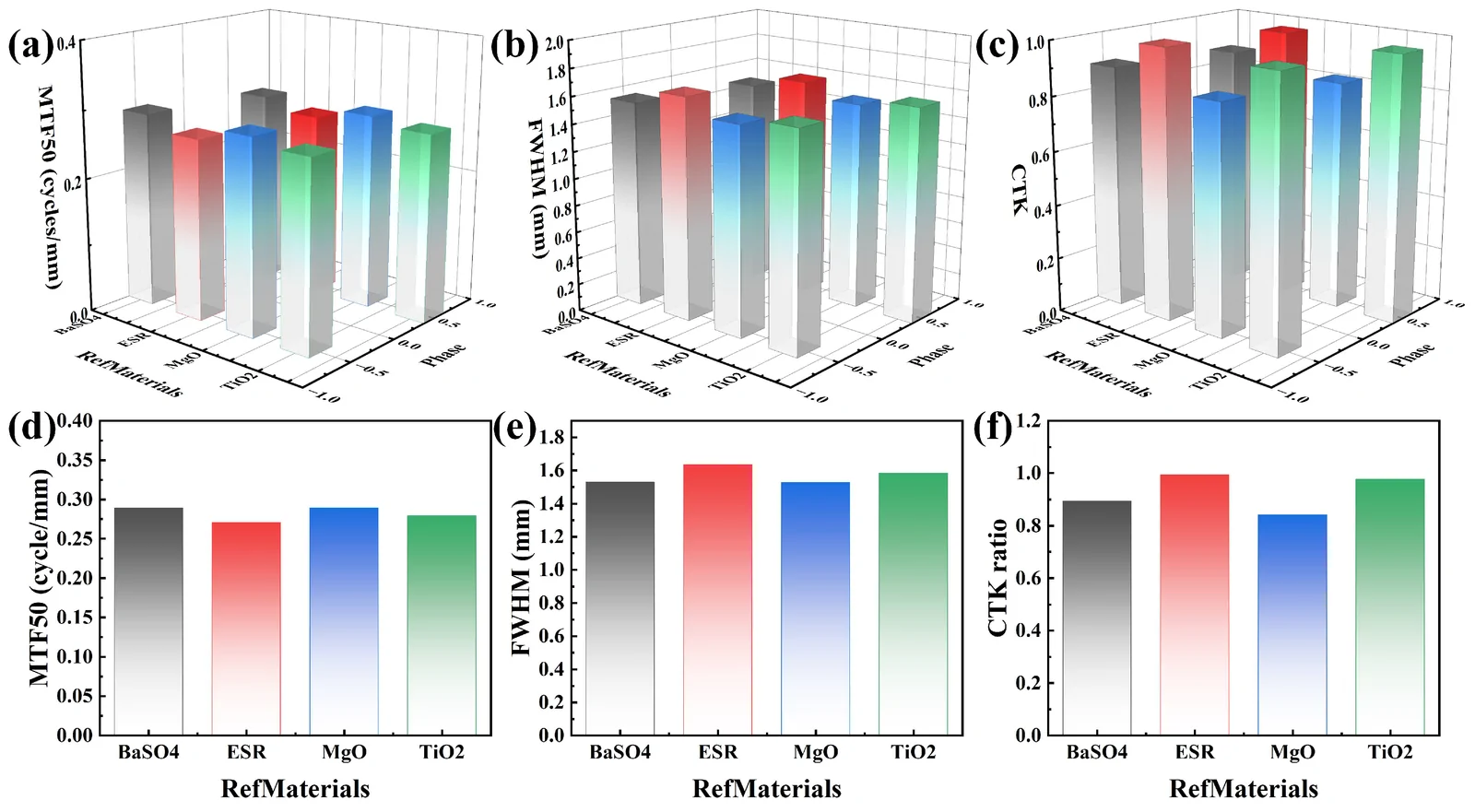
Response at phase = ±0.5 for TiO_2_, BaSO_4_, MgO, and ESR with PT = 1.0 mm and REF = 0.3 mm: (**a**–**c**) values at −0.5 and +0.5; (**d**–**f**) mean (±0.5) MTF50, FWHM, and neighboring-to-dominant-channel response ratio (*CTK*_ratio_).

Figure 5 compares the responses at *ϕ* = −0.5 and +0.5 and their mean. The mean neighboring-to-dominant-channel response ratios are 0.8404 for MgO, 0.8924 for BaSO_4_, 0.9759 for TiO_2_ and 0.9932 for ESR. MgO also gives the highest boundary MTF50 and the lowest boundary FWHM among the tested reflectors. These channel-response ratios quantify readout sharing under the specified illumination geometry; they should not be interpreted as the fractions of optical photons physically transported across the reflector interface [22,25,27].

### 3.4. Effect of the Metal Interlayer

**Figure 6:**
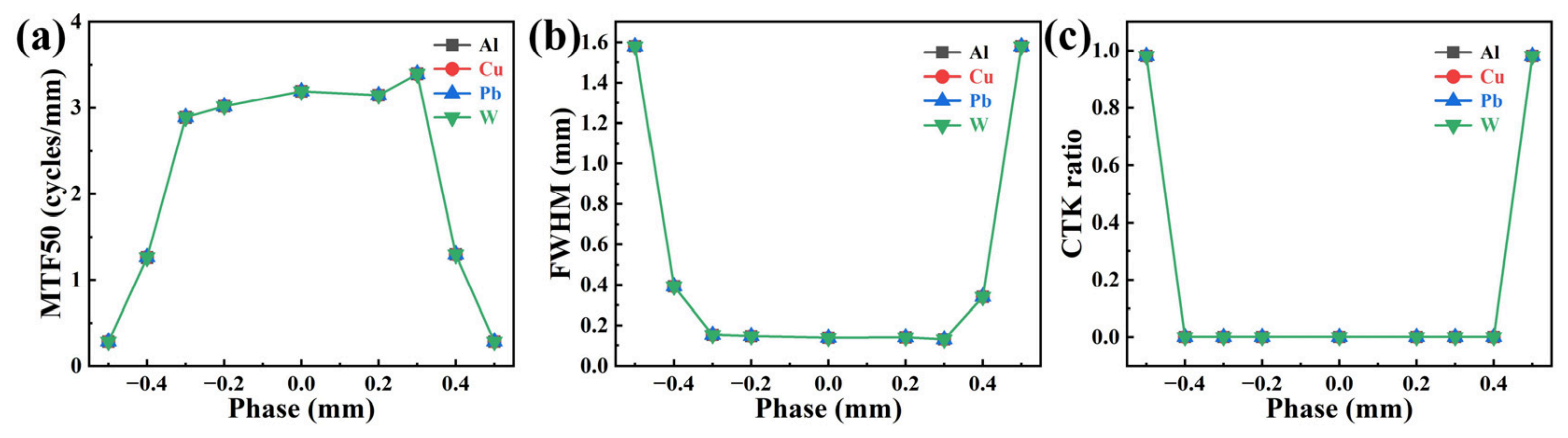
Phase-dependent response for Al, Cu, Pb, and W interlayers at PT = 1.0 mm, REF = 0.3 mm, MgO reflector, and 0.10 mm metal thickness: (**a**) MTF50; (**b**) FWHM; (**c**) optical crosstalk ratio.

Figure 6 shows the phase–response curves for different metal interlayer materials (Al, Cu, Pb, and W) under fixed conditions of PT = 1.0 mm, REF = 0.3 mm, MgO as the reflector material, and a metal interlayer thickness of 0.10 mm. It can be seen that the three sets of phase–response curves for the four metals almost completely overlap. No identifiable systematic differences are observed in MTF50, FWHM, or CTK ratio, either in the central region around phase ≈ 0 or in the boundary region around phase ≈ ±0.5. This indicates that, within the optical model and geometric configuration established in this work, the metal interlayer material itself is not a dominant factor governing spatial-resolution performance or optical crosstalk behavior [11,24]. The reason is that, in the present model, the position response is mainly determined by scintillation-light transport within the crystal, the reflection and scattering processes at the crystal–reflector interface, and the photon distribution returning to the readout side, whereas the metal interlayer does not substantially alter this dominant transport chain [13,23].

**Figure 7:**
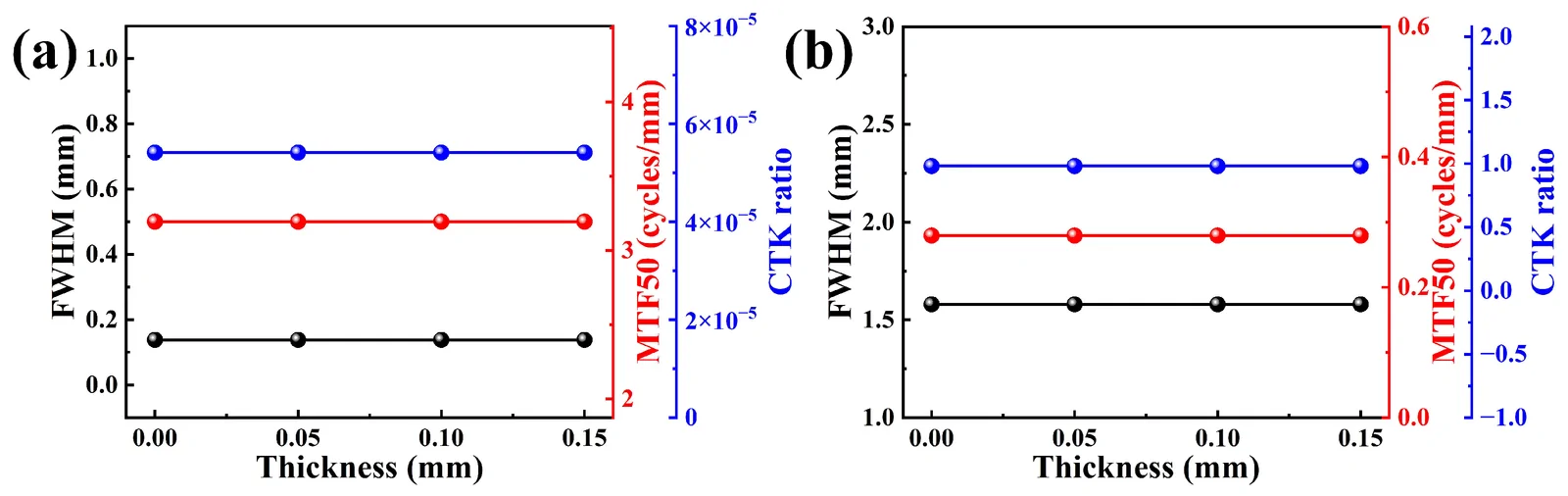
Effect of Pb interlayer thickness (0–0.15 mm) at PT = 1.0 mm, REF = 0.3 mm, and MgO reflector: (**a**) FWHM, MTF50, and optical crosstalk ratio at phase = 0; (**b**) the same metrics for mean (±0.5).

Figure 7 shows the variation in spatial-response metrics as the metal interlayer thickness increases from 0 to 0.15 mm, with MgO fixed as the reflector material and Pb fixed as the metal interlayer material. Figure 7a corresponds to the central-incidence condition, and Figure 7b corresponds to the boundary-averaged condition, mean (±0.5). As shown in the figure, neither the central FWHM, central MTF50, and central CTK ratio nor the corresponding metrics under the boundary-averaged condition exhibits any clear systematic variation with increasing metal interlayer thickness. This indicates that, in the 16 × 1 linear scintillator configuration investigated in this work, changes in the thickness of the metal septa do not significantly affect the optical-sharing statistics that determine spatial-resolution performance [11,23]. For central events, photons propagate mainly within the primary pixel and are collected by the readout side, so the metal layer imposes almost no additional constraint on their transport paths. For boundary events, although the position response is more sensitive to inter-pixel sharing, the width of the sharing band and the response gradient are still governed mainly by the reflection phase function at the reflector interface and the lateral transport correlation length near the boundary, rather than by the geometric thickening of the metal layer itself [13,24,30]. Therefore, within the scope of this study, the metal interlayer is better treated as a secondary optimization variable rather than a primary design degree of freedom for spatial-resolution performance [30].

### 3.5. ASG Geometry and Neighboring-Pixel Response Under the Scattering-Phantom Condition

Section 3.1, Section 3.2, Section 3.3 and Section 3.4 address scintillation-light transport after energy deposition. The ASG is positioned on the incident side and suppresses large-angle external scatter before energy deposition by limiting the angular acceptance. The internal geometry selected above was, therefore, fixed during the ASG scans, so the ASG acts as a complementary system-level component rather than as part of the reflector-controlled optical boundary.

**Figure 8:**
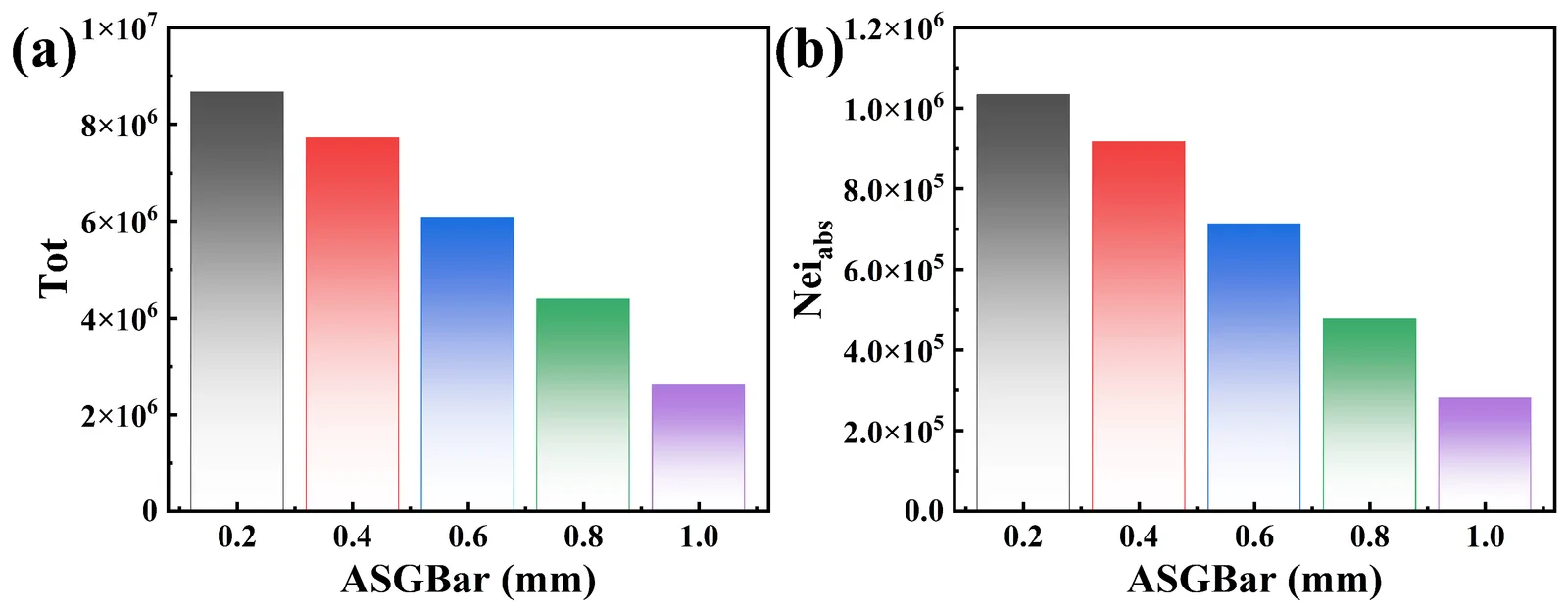
Effect of W ASG bar thickness (0.2–1.0 mm) under the HDPE scattering-phantom condition (PT = 1.0 mm, REF = 0.3 mm, MgO, 0.10 mm Pb interlayer, ASG height = 10 mm): (**a**) total positive-energy-deposition step records, Tot; (**b**) step records in neighboring channels 7 and 9, *Nei*_abs_. These are Screen-tally quantities, not independently classified scattered-photon detections.

As shown in Figure 8, increasing the W ASG bar thickness from 0.2 to 1.0 mm decreases the recorded Screen-step total, Tot, from 8.66 × 10^6^ to 2.60 × 10^6^ and the neighboring-step tally, Neiabs, from 1.033 × 10^6^ to 2.795 × 10^5^. The corresponding Neiabs/Tot fraction changes only from 0.1193 to 0.1077 (Figure S3), a decrease of 1.16 percentage points. The 72.9% reduction in neighboring-step records accompanies a 70.0% reduction in total step records. Since several deposited-energy steps can arise from one primary history, these step-count reductions must not be equated with changes in independent detected-photon counts, imaging sensitivity, or scatter-to-primary ratio [31,32,33,34].

With the 0.2 mm bar as the reference, the descriptive Screen-step retention factors *R*_Tot_ and *R*_Nei_ were calculated from the ratios of total and neighboring-channel step records, respectively:

(23) $$R_{\mathrm{Tot}}(b)=\frac{\mathrm{Tot}(b)}{\mathrm{Tot}(b_{0})},\;\;\;R_{\mathrm{Nei}}(b)=\frac{{\mathrm{Nei}}_{\mathrm{abs}}(b)}{{\mathrm{Nei}}_{\mathrm{abs}}(b_{0})}$$

Multiple Screen entries may originate from the same incident photon history and are, therefore, statistically correlated. Consequently, the ratios of Screen-step tallies are not equivalent to the ratios of independent detected events and are not used to infer imaging noise or compensating exposure time.

The dimensionless retention factors *R*_Tot_ and *R*_Nei_ describe, respectively, the total and neighboring-channel step tallies relative to the 0.2 mm bar configuration. Since the incident radiation was not classified by its scattering history in these tallies, a scatter-to-primary ratio (SPR) cannot be calculated. The image contrast-to-noise ratio (CNR) likewise requires defined signal and background regions and the associated noise statistics, which are not provided by these tallies.

At a bar thickness of 0.60 mm, *R*_Tot_ = 0.701 and *R*_Nei_ = 0.690: relative to 0.2 mm, the neighboring-step tally decreases by 31.0%, while the total step tally decreases by 29.9%. At 1.0 mm, the corresponding retention factors are 0.300 and 0.271. The 0.60 mm setting was retained as a conditional compromise in recorded-step counts over the sampled discrete geometries, not as a statistically verified or universally optimal ASG thickness.

At 0.60 mm bar thickness and 10 mm ASG height, *Nei*_frac_ = 0.117360. Primary-history bootstrap analysis gives a standard error of 0.004113 and a 95% confidence interval of 0.109227–0.125461 for this fraction (Table S3). The corresponding value at 0.2 mm is 0.119271. The 0.001911 difference between these point estimates is smaller than the standard error of the 0.60 mm value; however, the statistical significance of the difference cannot be established without a matched uncertainty estimate for the 0.2 mm simulation. These results support a trade-off between the step-based neighboring-channel tally and the total tally, rather than a demonstrated SPR or CNR improvement.

**Figure 9:**
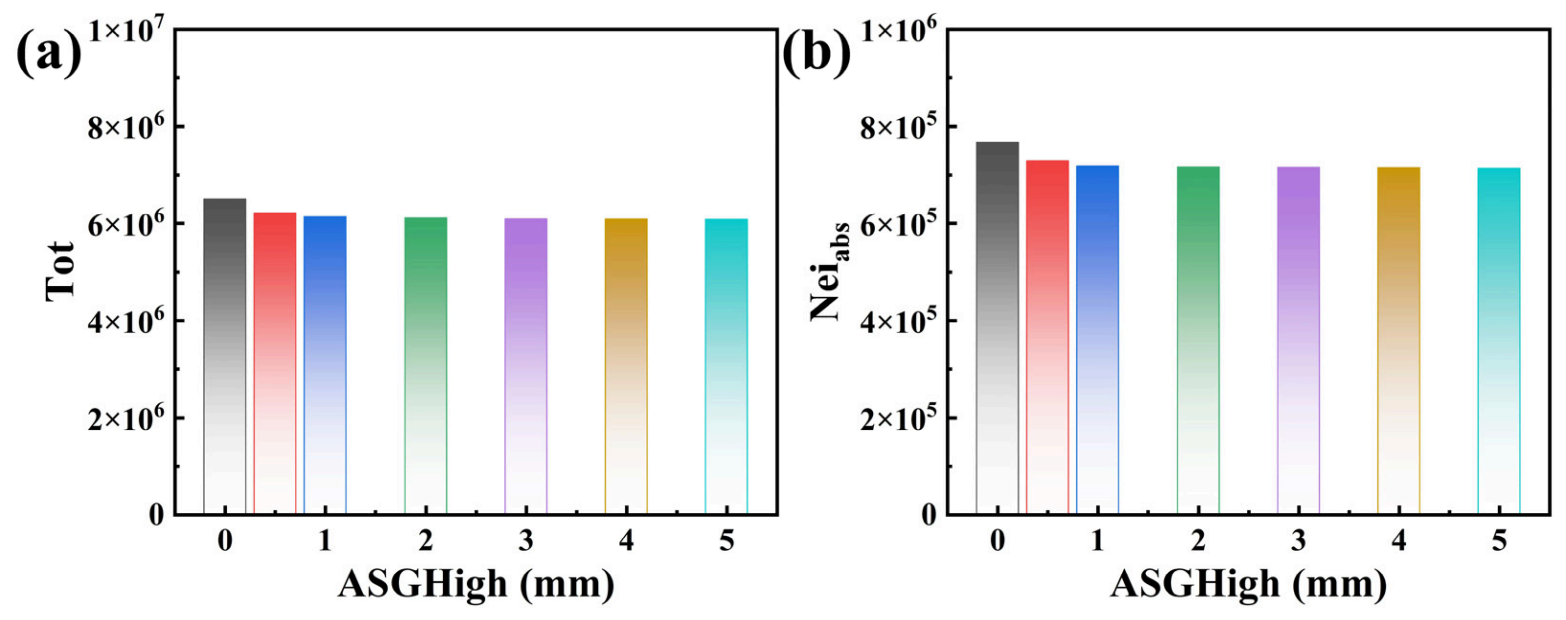
Effect of W ASG height (0.2–5.0 mm) at a fixed bar thickness of 0.60 mm under the HDPE scattering-phantom condition: (**a**) total positive-energy-deposition step records, Tot; (**b**) Screen-step records in channels 7 and 9, *Nei*_abs_.

Figure 9 shows that increasing the ASG height from 0.2 to 5.0 mm at a fixed 0.60 mm bar thickness changes Tot from approximately 6.50 × 10^6^ to 6.08 × 10^6^ (−6.5%) and Neiabs from approximately 7.67 × 10^5^ to 7.13 × 10^5^ (−7.0%). These are changes in correlated energy-deposition-step records, not independently counted primary detections. The 5.0 mm height had the smallest absolute neighboring-step tally in the sampled range and was retained as a conditional geometric choice. A demonstration of improved SPR, image CNR, or dose efficiency would require separately identified primary/scattered events and task-specific image statistics.

### 3.6. Quantitative Image-Level Evaluation

Following the procedure in Section 2.3, PT = 1.0 mm was selected because increasing PT from 1.0 to 1.2 mm improved the central FWHM and MTF50 by only 4.2% and 4.4%, respectively, while the worst-case FWHM increased from 1.8168 to 2.0339 mm and the worst-case MTF50 decreased from 0.2429 to 0.2170 cycles mm^−1^. With PT fixed at 1.0 mm, REF = 0.3 mm gave the best boundary FWHM and MTF50 without degrading the central response, and MgO gave a favorable balance across the central and boundary response metrics.

**Figure 10:**
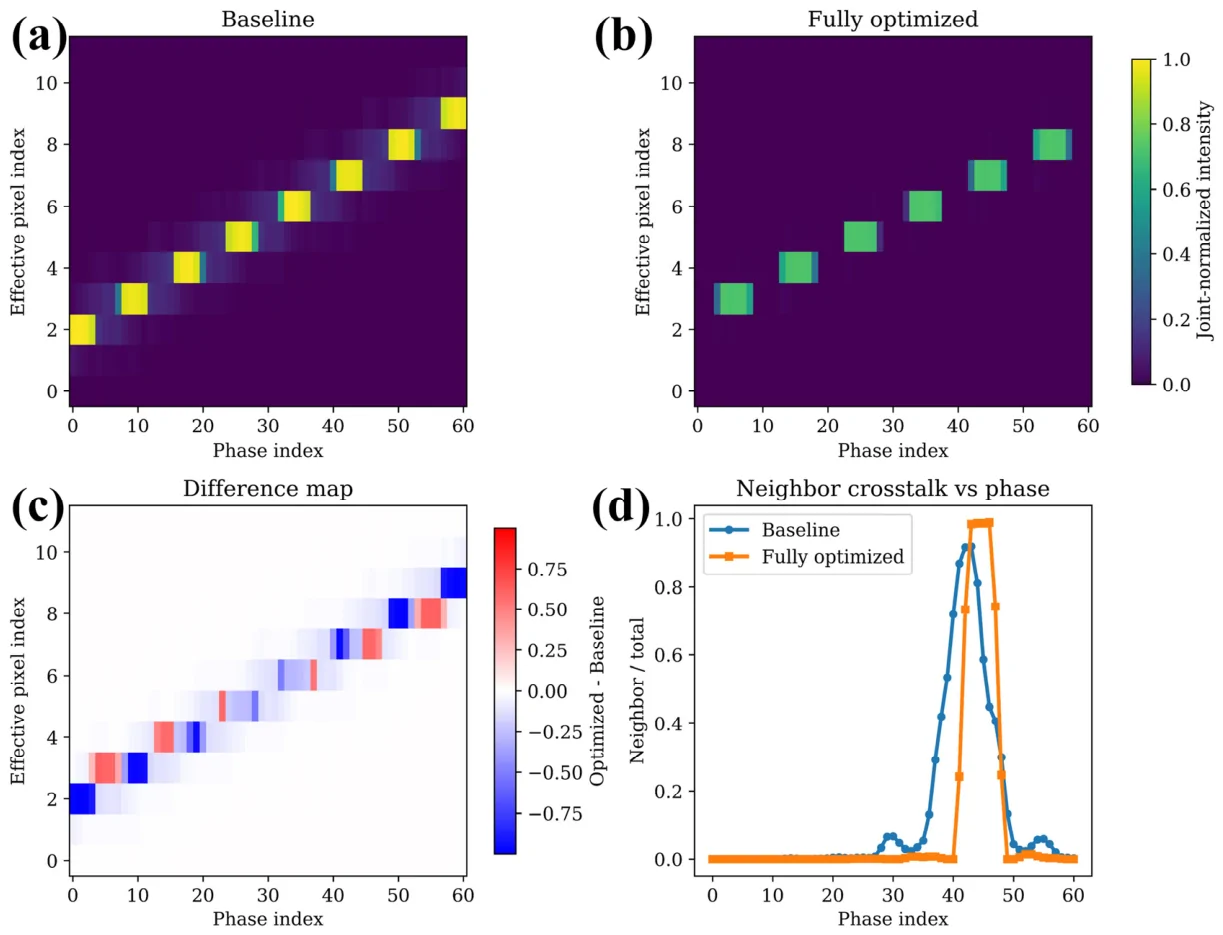
Phase–pixel response comparison for the baseline and optimized configurations defined in Table 1 during narrow-strip scanning across pixel boundaries: (**a**) baseline configuration; (**b**) optimized configuration; (**c**) difference map (optimized − baseline); (**d**) neighboring-pixel response as a function of scan phase.

**Figure 11:**
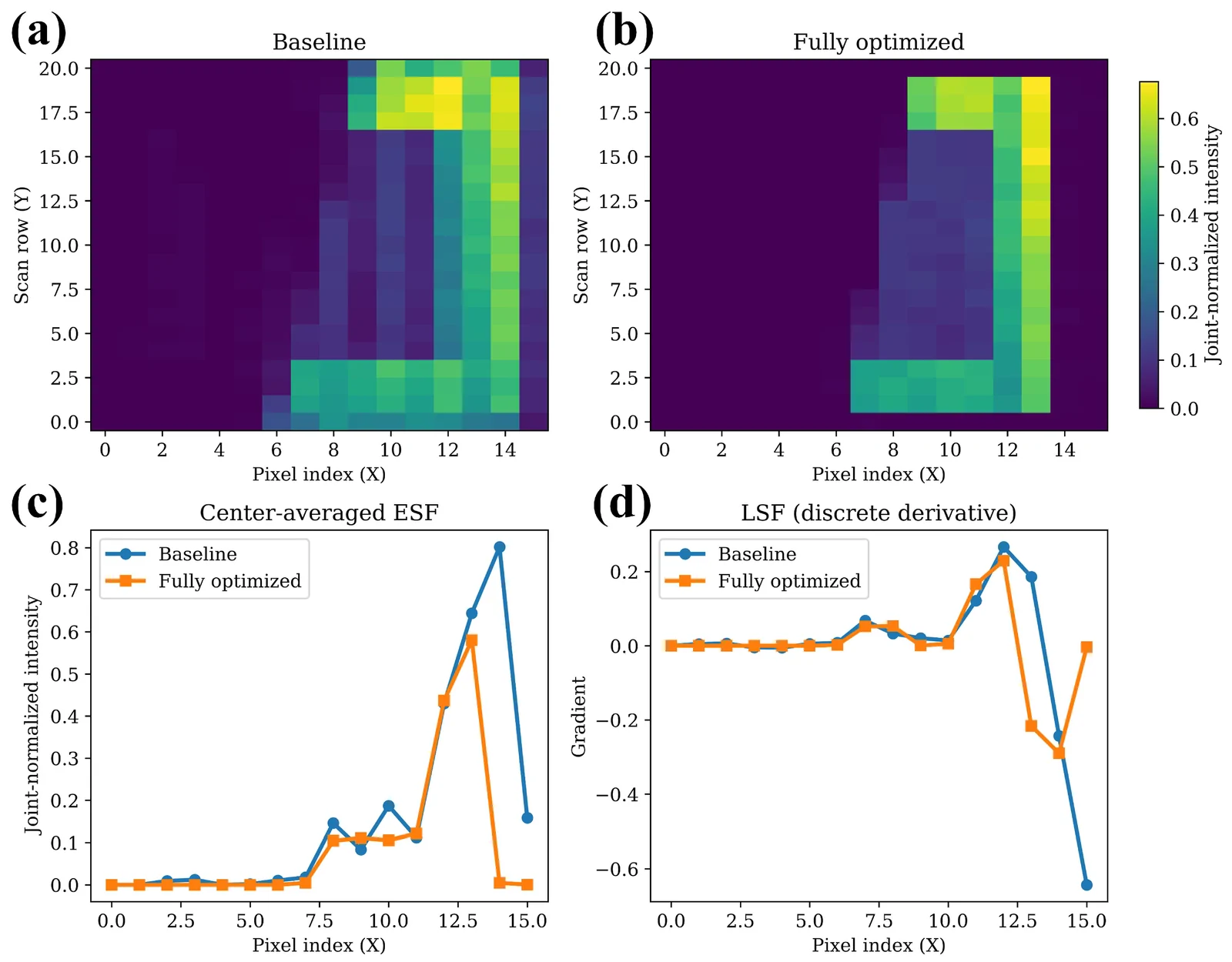
Slanted-edge comparison under the strong-scattering condition for the baseline and optimized configurations defined in Table 1: (**a**) baseline image; (**b**) optimized image; (**c**) center-averaged ESF; (**d**) LSF obtained by discrete differentiation.

The metal-interlayer scans showed no systematic material or thickness dependence, so a 0.10 mm Pb interlayer was retained as a representative setting. The W ASG with 0.60 mm bars, a height of 5.0 mm, and a placement interval of one pixel pitch was selected from the conditional step-count trade-off in Section 3.5. Table 1 defines the baseline and optimized configurations used in Figure 10 and Figure 11. Within each imaging task, the source, primary histories, scoring method, and reconstruction procedure were identical between the two configurations except for the detector parameters listed in Table 1.

In Figure 10, both configurations were irradiated using an 80 keV narrow-strip source at z = −150 mm. The strip size was 0.10 × 16 mm^2^, and 61 equally spaced positions from x = −4 to 4 mm were simulated with 500 primary events per position; no HDPE scatterer was used. In Figure 11, an 80 keV source at z = −150 mm and a 100 mm thick HDPE scatterer positioned 5 mm in front of the detector were used. The 12° slanted edge was sampled at 21 Y positions using a 16 × 0.04 mm^2^ strip source with 1000 primary events per position.

The optimized configuration in Figure 10 and Figure 11 includes the ASG listed in Table 1. Figure 10 evaluates the phase response of the complete geometry without the HDPE scatterer, whereas Figure 11 evaluates the same geometry under strong-scattering conditions. By contrast, the ASG was disabled and PT was fixed at 1.0 mm in Figures S4 and S5 to isolate the effects of the reflector and metal-interlayer parameters on the internal optical response.

Compared with the baseline configuration, the optimized configuration in Figure 10 shows a narrower main response band and weaker lateral tails. Across all 61 scan positions, the phase-integrated adjacent-channel response fraction decreases from 13.66% (95% bootstrap interval, 12.39–14.78%) to 1.53% (1.17–1.94%), corresponding to an 88.8% reduction. The optimized-minus-baseline difference is −12.13 percentage points (95% interval, −13.27 to −10.80). The total recorded optical-photon count decreases from 4.083 × 10^7^ to 2.155 × 10^7^, corresponding to a 47.2% reduction (95% bootstrap interval, 45.5–48.8%). The selected geometry, therefore, improves response localization while reducing recorded photon throughput.

The total recorded optical-photon response in the phase scan decreased from 4.083 × 10^7^ to 2.155 × 10^7^, corresponding to a relative light-throughput factor of 0.528. If these totals were proportional to independent detected-photoelectron statistics under otherwise-identical conditions, the statistical contribution proportional to *N*^−1/2^ would increase by a factor of (4.083/2.155)^1/2^ ≈ 1.376 (37.6%). This is illustrative photon-statistical scaling, not a prediction of energy resolution or absolute detection sensitivity. The phase-integrated tallies do not provide per-event photoelectron yields or account for detector gain and electronic noise; consequently, the spatial-response improvement does not establish an improvement in energy resolution or sensitivity.

The slanted-edge results in Figure 11 show a narrower edge transition and lower residual response in the dark region for the optimized configuration. Error-function fitting gives 10–90% edge-spread widths of 2.951 and 1.876 mm for the baseline and optimized configurations, respectively, a reduction of 36.4%. The imaging MTF50 point estimates are 0.163 and 0.256 cycles mm^−1^ for the baseline and optimized configurations, respectively [35,36]. Bootstrap refitting and cumulative-history diagnostics showed substantial fit instability, so these values are reported descriptively rather than as evidence of a statistically significant difference or convergence (Table S5 and Figure S6b).

Figures S4 and S5 use ASG-free auxiliary configurations with PT fixed at 1.0 mm. The reference optical configuration uses REF = 0.5 mm, TiO_2_, and no metal interlayer; the optimized optical configuration uses REF = 0.3 mm, MgO, and a 0.10 mm Pb interlayer. The Siemens-star images in Figure S4 provide a qualitative two-dimensional comparison of these optical configurations. The one-dimensional bar pattern in Figure S5 provides a quantitative comparison aligned with the detector geometry. The modulation increases from 0.361 for the reference optical configuration to 0.668 for the optimized optical configuration, corresponding to an 85.2% improvement. The lower absolute peaks in the optimized case are accompanied by a larger reduction in the nominally dark bars, producing greater bright–dark separation [28,29,37].

Taken together, Figure 10, Figure 11, Figure S4 and Figure S5 and Table 2 show that the internal optical structure and ASG act at different stages. PT, REF, and the reflector affect scintillation-light response sharing after energy deposition, whereas the ASG changes radiation acceptance before energy deposition. The ASG Screen-step tallies describe the geometry-dependent response under the modeled scattering phantom but do not by themselves separate scattered from unscattered incident radiation. The ASG-free supplementary tests isolate the internal optical contribution.

### 3.7. Scope and Limitations of the Simulation Study

This study assesses simulated performance under the specified geometry, irradiation, optical-surface and reconstruction assumptions. A wavelength-dependent optical-surface EFFICIENCY was assigned to the equivalent SiPD interface. Optical photons were tallied at this interface and used as relative channel-response weights, without an explicit optical-boundary detection check or conversion to calibrated photoelectrons. The model does not describe SiPM gain fluctuations, dark noise, electronic readout, saturation or threshold discrimination. Bootstrap intervals are reported for the ASG Screen-step fraction (Table S3) and the phase-integrated optical response (Table S4). The slanted-edge MTF50 estimates are descriptive because resampled fits showed substantial variability (Table S5). Event-level records for the original parameter scans were unavailable for uncertainty estimates of FWHM, Gaussian-equivalent MTF50 and local response ratios; independent-seed convergence across all configurations was not assessed. The step-based ASG scoring does not separately identify primary and scattered histories; consequently, detector-level energy resolution, SPR, CNR, absolute sensitivity and dose efficiency were not evaluated. Experimental measurements and task-specific simulations are required to assess these system-level quantities.

The reported choice of PT = 1.0 mm, REF = 0.3 mm, MgO and a W ASG with 0.60 mm bar thickness and 5.0 mm height applies to the sampled discrete parameter combinations. Because the 60 keV structural scans and the 80 keV imaging and ASG calculations do not constitute a controlled energy dependence study, the stability of this choice under different photon energies, incidence angles or scattering conditions has not been established.

## 4. Conclusions

This Geant4 study examined the effects of pixel geometry, reflector properties, and ASG dimensions on a 16 × 1 Ce:GAGG linear scintillator detector. Under the modeled optical conditions, PT = 1.0 mm, REF = 0.3 mm, and MgO gave a favorable compromise between the central and boundary position responses, whereas the tested metal interlayers showed little dependence in the evaluated optical metrics. An ASG with 0.60 mm W bars and a height of 5.0 mm was selected from the simulated trade-off between total and neighboring-channel energy-deposition-step tallies; the present scoring does not establish an improvement in SPR or CNR. The phase-integrated adjacent-channel optical-response fraction decreased from 13.66% to 1.53%; the slanted-edge 10–90% transition width decreased from 2.951 to 1.876 mm; and the imaging MTF50 point estimate increased from 0.163 to 0.256 cycles mm^−1^. In the ASG-free optical comparison, bar-pattern modulation increased from 0.361 to 0.668. These comparisons are specific to the simulated geometry, optical inputs, and irradiation conditions.

## Figures and Tables

**Table 1:** Structural and optical parameters of the baseline and optimized configurations used for image-level evaluation in Figure 10 and Figure 11.

Parameter	Baseline Configuration	Optimized Configuration
Array format	16 × 1	16 × 1
Ce:GAGG pixel dimensions, X × Y × Z (mm^3^)	0.6 × 16 × 8	1.0 × 16 × 8
Pixel dimension along X, PT (mm)	0.6	1.0
Reflector-layer thickness, REF (mm)	0.5	0.3
Effective pixel pitch, p = PT + REF (mm)	1.1	1.3
Reflector material	TiO_2_	MgO
Reflector density (g·cm^−3^)	4.23	3.58
Optical-surface type and model	dielectric–dielectric, UNIFIED	dielectric–dielectric, UNIFIED
Surface finish	polishedfrontpainted	groundfrontpainted
Surface-roughness parameter, σ^α^(rad)	0.15	0.25
Reflector refractive index, 1.5–2.8 eV	2.60–2.70	1.74
Reflectivity, 1.5–2.8 eV	0.98	0.96–0.98
SPECULARLOBE/SPECULARSPIKE/BACKSCATTER	0.80/0.05/0.15	0.75/0.05/0.20
Metal-interlayer material	Pb	Pb
Metal-interlayer thickness (mm)	0	0.10
ASG material	W	W
ASG bar thickness (mm)	0	0.60
ASG height (mm)	0	5.0
ASG placement interval	0	One pixel pitch
Equivalent SiPD-layer thickness (mm)	0.075	0.075
Top-cover thickness (mm)	0.05	0.05
Top-cover material	TiO_2_	MgO

Note: A value of zero indicates that the corresponding metal interlayer or ASG component was not included. The common Ce:GAGG properties are given in Section 2.1.

**Table 2:** Quantitative results of the image-level evaluation.

Imaging Task and Metric	Baseline or Reference Configuration	Optimized Configuration	Relative Change
Phase scan: phase-integrated adjacent-pixel crosstalk, *CTK*_int_ (%)	13.66	1.53	−88.8%
Phase scan: total recorded optical-photon count (×10^7^)	4.083	2.155	−47.2%
Slanted edge: 10–90% edge-spread width, *W*_10–90_ (mm)	2.951	1.876	−36.4%
Slanted edge: imaging MTF50 (cycles mm^−1^)	0.163	0.256	+57.3%
One-dimensional bar pattern: modulation, *M*	0.361	0.668	+85.2%

Note: The phase-scan and slanted-edge results use the complete configurations in Table 1. The bar-pattern test uses the ASG-free optical configurations defined in the text. Relative change is calculated as (optimized − baseline or reference)/(baseline or reference) × 100%.

## Data Availability

The original contributions presented in this study are included in the article/Supplementary Materials. Further inquiries can be directed to the corresponding authors.

## Supplementary Materials

The following supporting information can be downloaded at: https://www.mdpi.com/article/10.3390/ma19214516/s1, Figure S1. Comparison of the LSF and MTF between the published results and the present simulation for code and method consistency assessment: (a) literature LSF; (b) literature MTF [21]; (c) simulated LSF; (d) simulated MTF. Figure S2. Two-dimensional PT–phase response maps with REF = 0.5 mm and TiO_2_ fixed: (a) MTF50; (b) FWHM. Figure S3. Effect of W ASG bar thickness on the scatter-induced neighboring-pixel count fraction, *Nei*_frac_, under the strong-scattering condition. Figure S4. Siemens-star comparison for ASG-free configurations with PT = 1.0 mm: (a) reference optical configuration (REF = 0.5 mm, TiO_2_, no metal interlayer); (b) optimized optical configuration (REF = 0.3 mm, MgO, 0.10 mm Pb); (c) difference map (optimized − reference). Figure S5. One-dimensional bar-pattern comparison for ASG-free configurations with PT = 1.0 mm: (a) reference optical configuration; (b) optimized optical configuration; (c) response profiles; (d) pixel-wise difference. The modulation values are 0.361 and 0.668, respectively. Figure S6. Statistical uncertainty and sampling-depth diagnostics for the image-level comparisons. (a) Phase-integrated adjacent-channel optical-response fraction (CTKint) for the baseline and optimized configurations in Figure 10. Points denote the estimates from 500 primary histories at each of 61 scan positions; error bars show 95% percentile confidence intervals from 2000 bootstrap replicates. Primary EventIDs were resampled together across scan positions within each configuration, and the two configurations were resampled independently. (b) Imaging MTF50 from the slanted-edge fit in Figure 11, recalculated using cumulative subsets of 250, 500, 750 and 1000 primary histories per scan position. Blue circles and orange squares denote the baseline and optimized configurations, respectively. The central seven scan rows were used at each sampling depth. Lines connect the estimates as visual guides. The subsets are nested and do not constitute independent random-seed convergence tests. Table S1. Material and optical-surface parameters used for the reflector and top-cover layers. Table S2. Geant4 physics configuration, source parameters and primary-event statistics for the investigated simulation tasks. Table S3. Event-level statistical analysis of the neighboring-channel Screen-step fraction for the W ASG with 0.60 mm bar thickness and 10 mm height. Table S4. Primary-history bootstrap uncertainty in the phase-integrated optical response for the configurations in Figure 10. Table S5. Slanted-edge fit-stability and cumulative-history diagnostics for the configurations in Figure 11. A. Bootstrap fit-stability diagnostics. B. Cumulative-history diagnostic.
