# Supplementary material for: Multi-Parameter Optimization and Imaging Performance Evaluation of a 16 × 1 Ce:GAGG Linear Scintillator Detector: A Monte Carlo Simulation Study
Source: Materials (Basel). 2026 Oct 27;19(21):4516. doi: 10.3390/ma19214516 (PMC13764406; doi:10.3390/ma19214516)
Supplement: Supplementary file 1 [file materials-19-04516-s001.zip › materials-4599067-supplementary.pdf]

Multi-Parameter Optimization and Imaging Performance Evaluation of a 16 × 1 Ce:GAGG Linear Scintillator Detector: A Monte Carlo Simulation Study

Zhu Liu [1,†], Yanggang Jia [1,†], Zhaoran Guan [1], Xinya Wang [1], Meng Cao [1,2,*], Haofei Huang [1,*], Linjun Wang [1,2,3,*] and Jian Huang [1,2,3]

[1] State Key Laboratory of Materials for Advanced Nuclear Energy & School of Materials Science and Engineering, Shanghai University, Shanghai 200444, China; monicazubek@163.com (Z.G.)
[2] Zhejiang Institute of Advanced Materials, Shanghai University, Jiaxing 314113, China
[3] Shanghai Engineering Research Center for Integrated Circuits and Advanced Display Materials, Shanghai University, Shanghai 200444, China
* Correspondence: caomeng@shu.edu.cn (M.C.); kardson@shu.edu.cn (H.H.); ljwang@shu.edu.cn (L.W.)
† These authors contributed equally to this work.

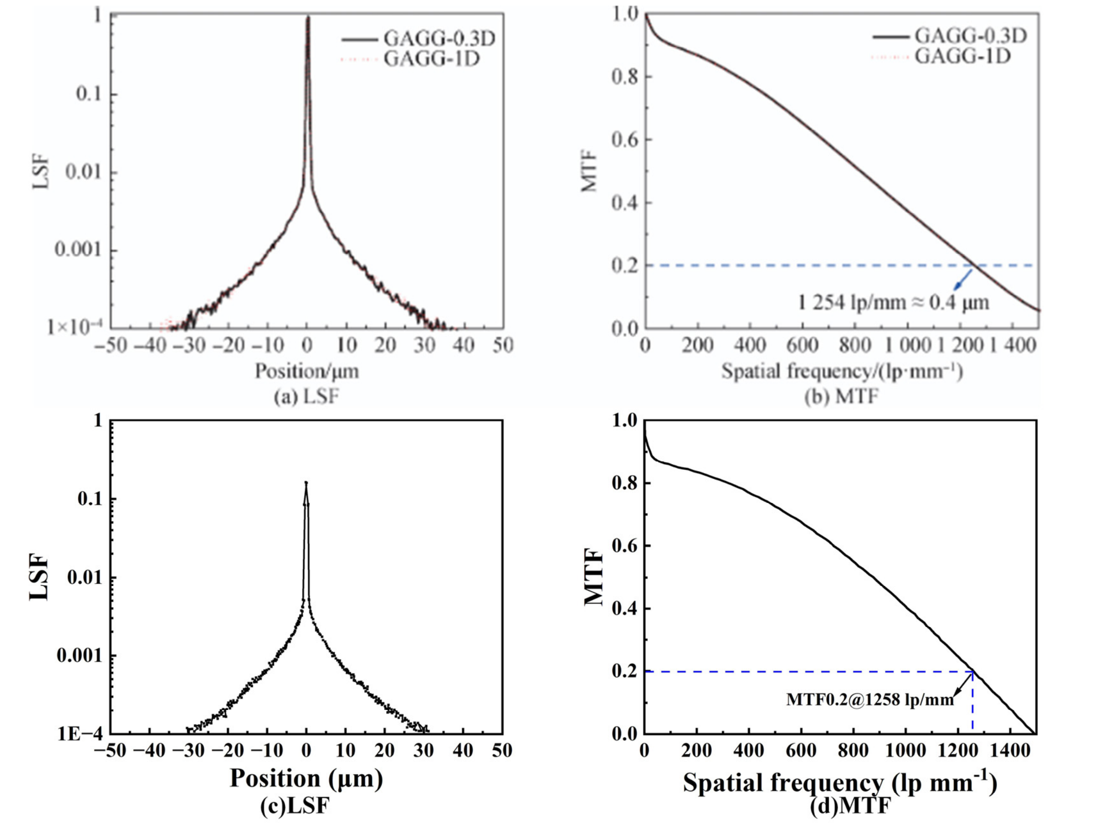

Fig. S1. Comparison of the LSF and MTF between the published results and the present simulation for code and method consistency assessment: (a) literature LSF; (b) literature MTF [21]; (c) simulated LSF; (d) simulated MTF.

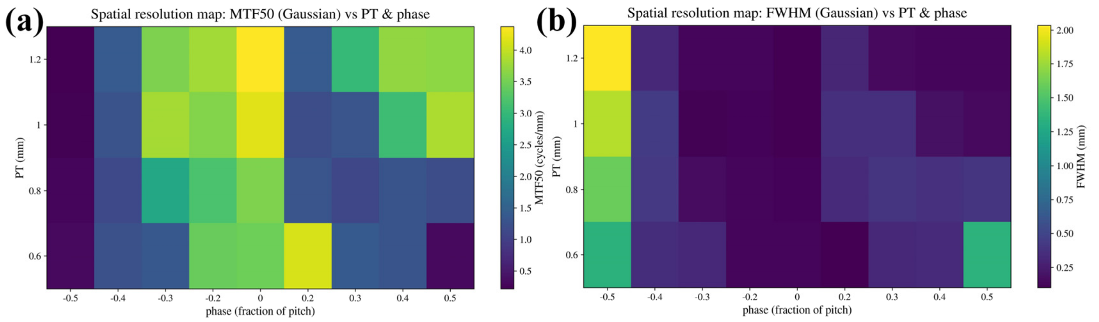

Fig. S2. Two-dimensional PT–phase response maps with REF = 0.5 mm and TiO_2 fixed: (a) MTF50; (b) FWHM.

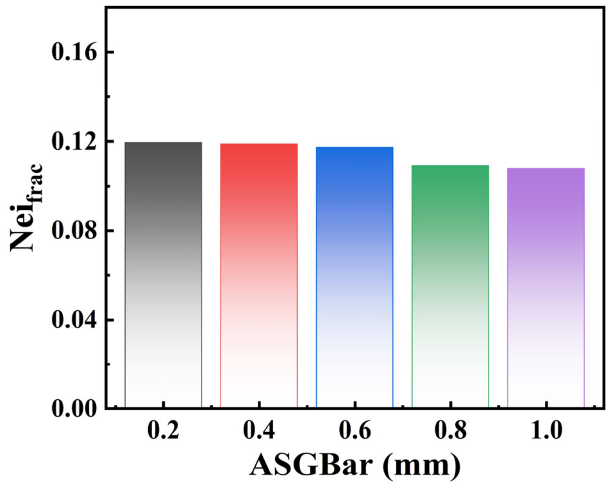

Fig. S3. Effect of W ASG bar thickness on the scatter-induced neighboring-pixel count fraction, Nei_{frac}, under the strong-scattering condition.

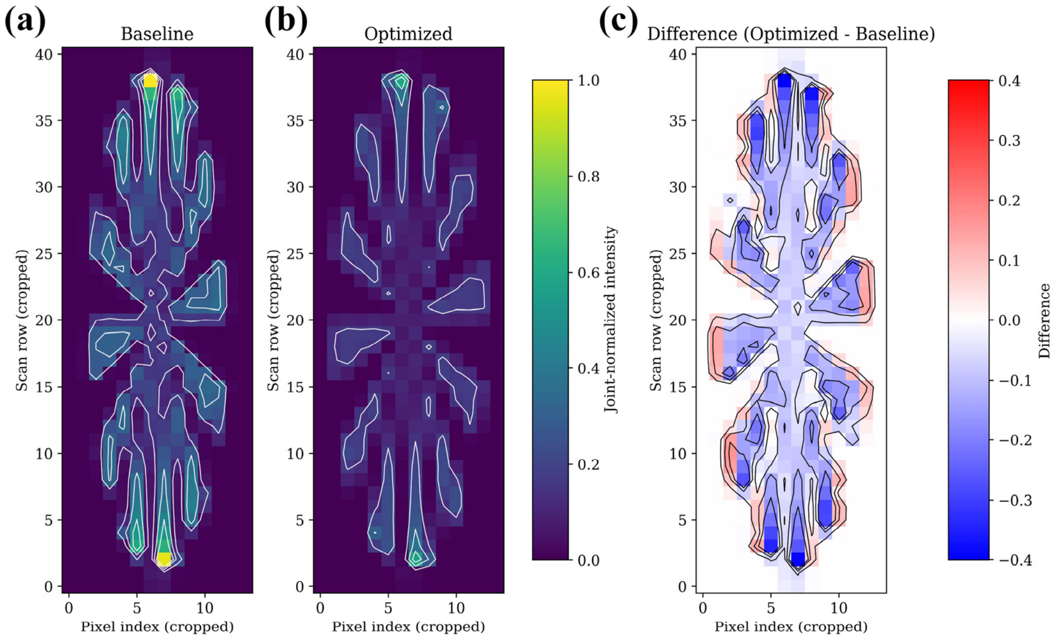

Fig. S4. Siemens-star comparison for ASG-free configurations with PT = 1.0 mm: (a) reference optical configuration (REF = 0.5 mm, TiO_2, no metal interlayer); (b) optimized optical configuration (REF = 0.3 mm, MgO, 0.10 mm Pb); (c) difference map (optimized − reference).

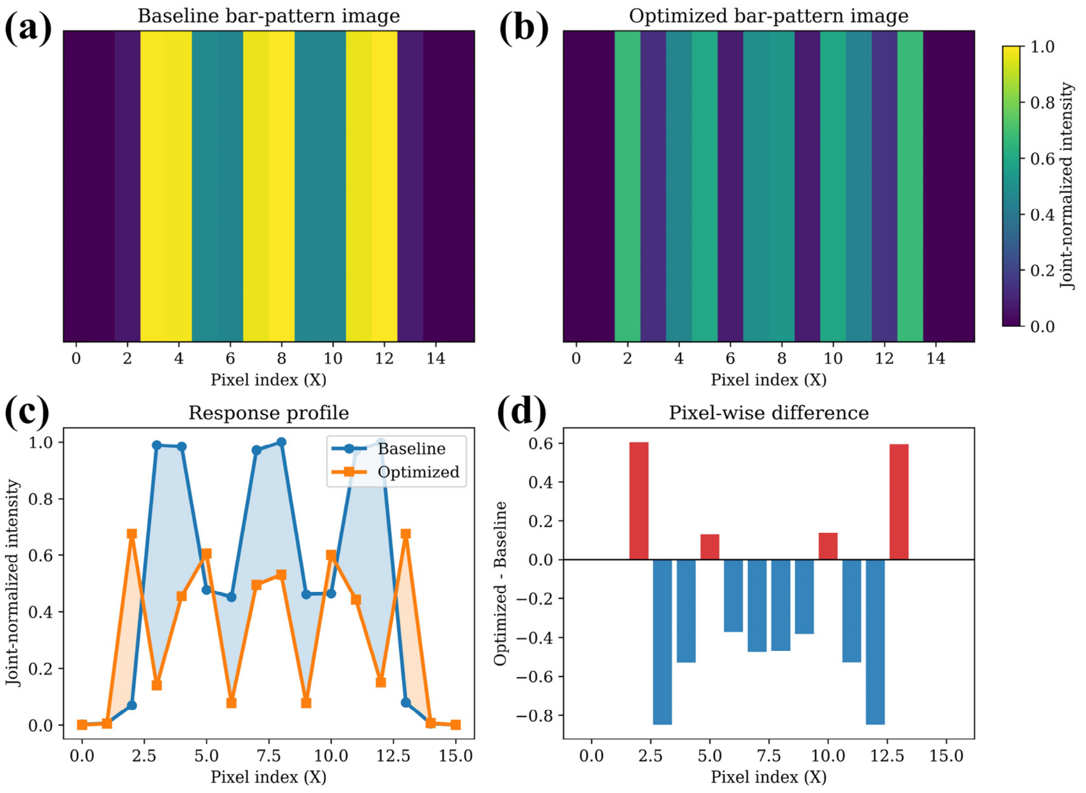

Fig. S5. One-dimensional bar-pattern comparison for ASG-free configurations with PT = 1.0 mm: (a) reference optical configuration; (b) optimized optical configuration; (c) response profiles; (d) pixel-wise difference. The modulation values are 0.361 and 0.668, respectively.

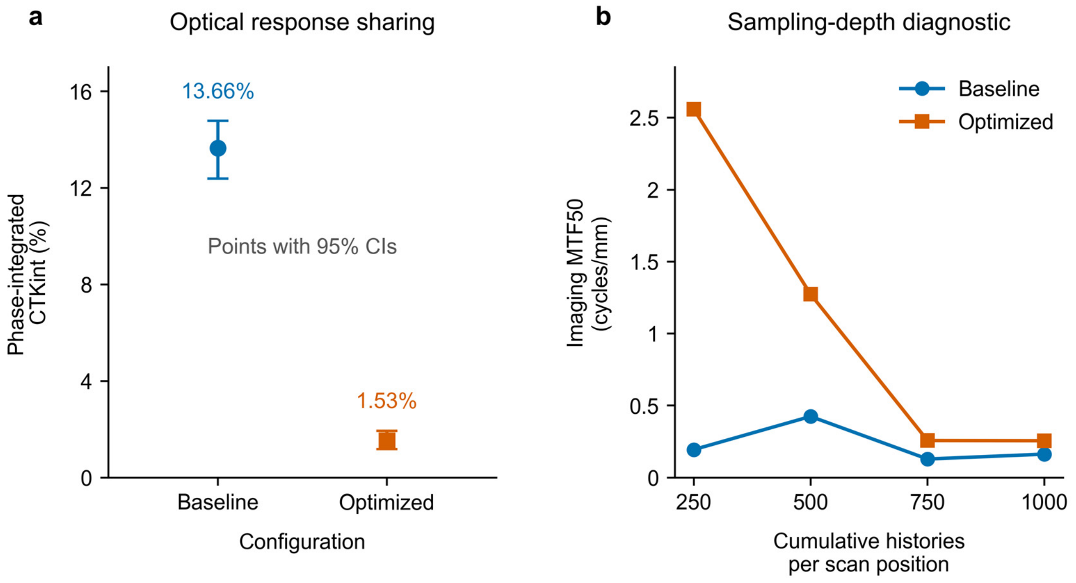

Fig. S6. Statistical uncertainty and sampling-depth diagnostics for the image-level comparisons. (a) Phase-integrated adjacent-channel optical-response fraction (CTKint) for the baseline and optimized configurations in Fig. 10. Points denote the estimates from 500 primary histories at each of 61 scan positions; error bars show 95% percentile confidence intervals from 2,000 bootstrap replicates. Primary EventIDs were resampled together across scan positions within each configuration, and the two configurations were resampled independently. (b) Imaging MTF50 from the slanted-edge fit in Fig. 11, recalculated using cumulative subsets of 250, 500, 750 and 1,000 primary histories per scan position. Blue circles and orange squares denote the baseline and optimized configurations, respectively. The central seven scan rows were used at each sampling depth. Lines connect the estimates as visual guides. The subsets are nested and do not constitute independent random-seed convergence tests.

Event-level statistical assessment of the image-response calculations

The archived ScoringPhotons records were aggregated by primary EventID and readout channel. Primary histories with no recorded optical photons were retained as zero-response histories. The channel sums reproduced the archived response matrices exactly. The phase scan contained 61 positions with 500 histories per position. The slanted-edge reanalysis used the central seven scan rows (indices 7–13), with 1,000 histories per row.

The archived launch scripts reused the default simulation seed of 345354. To retain possible dependence associated with this launch scheme, resampling was synchronized by EventID across scan positions within each configuration. The two configurations were resampled independently for the primary analysis. We generated 2,000 nonparametric bootstrap replicates using NumPy PCG64 with resampling seed 20261016. A sensitivity analysis synchronized the resampled EventID indices across configurations (seed 20261015). The resampling scheme does not identify same-numbered histories at different positions as the same physical photon.

For each replicate, the optical counts were summed by channel, and the dominant channel was selected anew at every scan position. CTKint, total recorded optical-photon count and between-configuration changes were then recalculated. Zero-response histories and zero-response positions were retained throughout. Standard errors are the sample standard deviations of the replicate estimates; 95% percentile intervals use the 2.5th and 97.5th percentiles (Table S4).

The reported slanted-edge point estimates were reproduced using the error-function model and interval stated in Section 2.4. In this reanalysis, the mean profile over rows 7–13 was normalized to its maximum, and pixel coordinates were $x_i = (i - 7.5)p$, with $p = 1.1$ or 1.3 mm. Fits used only $-2.0 \leqslant x \leqslant 7.2$ mm. Nonlinear least squares was initialized (background, amplitude, edge position, width) = (0, 1, 0 mm, 1 mm), with no parameter bounds and a maximum of 20,000 function evaluations. The fitted width was represented by $|\sigma|$. Every bootstrap sample was processed using this

same procedure.

All numerically returned fits were retained, including fits with covariance warnings or extreme widths. Table S5 reports the resulting bootstrap fit-stability ranges. These ranges reflect both resampling variability and weakly constrained nonlinear fitting and are not interpreted as validated performance confidence intervals. Cumulative-history diagnostics used the first 250, 500, 750 and 1,000 histories per row with the same analysis settings. These nested subsets assess sampling-depth sensitivity within the archived runs, not convergence across independent simulation seeds.

Table S1 lists the material and UNIFIED optical-surface parameters used for the reflector and top-cover layers. In each simulation case, the top cover used the same material and optical-surface definition as the selected reflector.

Table S1. Material and optical-surface parameters used for the reflector and top-cover layers.

Material	Density (g cm^{-3})	Surface finish	σ_a (rad)	Refractive index	Reflectivity	Specular lobe / spike / backscatter
TiO_2	4.23	polishedfrontpainted	0.15	2.70–2.60	0.98	0.80 / 0.05 / 0.15
$BaSO_4$	4.50	groundfrontpainted	0.30	1.64	0.980–0.985	0.85 / 0.01 / 0.14
MgO	3.58	groundfrontpainted	0.25	1.74	0.96–0.98	0.75 / 0.05 / 0.20
ESR	1.40	polishedfrontpainted	0.01	1.58	0.985–0.990	0.01 / 0.98 / 0.01

Note: All surfaces were defined as dielectric–dielectric using the UNIFIED model. Values were assigned at photon energies of 1.5, 1.8, 2.1, 2.25, 2.35, 2.5, and 2.8 eV. No independent bulk absorption or transmission coefficient was assigned to the top-cover layer.

Table S2. Geant4 physics configuration, source parameters and primary-event statistics for the investigated simulation tasks.

Simulation parameter	Value or setting
Geant4 version	11.2.2 (geant4-11-02-patch-02; multithreaded)
Hadronic and electromagnetic physics	FTFP_BERT with G4EmStandardPhysics_option4
Optical and additional physics	G4OpticalPhysics; G4DecayPhysics; G4RadioactiveDecayPhysics; 0.1 mm production cut; scintillation secondaries tracked first; Cherenkov emission, optical Rayleigh scattering and Mie scattering disabled
Ce:GAGG scintillation inputs	Density 6.62 g cm^{-3}; refractive index 1.9; effective optical absorption length 300 mm; light yield 46,000 photons MeV^{-1}; decay time 90 ns
PT spatial-response scan	Monoenergetic 60 keV photons; normally incident 0.04 × 16 mm^2 narrow strip; source z = −200 mm; nine irradiation positions; 200,000 primary photons per position
Fig. 10: phase–pixel response	Monoenergetic 80 keV photons; 0.10 × 16 mm^2 narrow strip; source z = −150 mm; 61 positions; 500 primaries per position; no HDPE scatterer
Fig. 11: slanted-edge imaging	Monoenergetic 80 keV photons; 12° edge; 16 × 0.04 mm^2 narrow strip; source z = −150 mm; 21 positions; 1,000 primaries per position; 100 mm HDPE scatterer; 5 mm gap

ASG bar-thickness comparison (Fig. 8)	W ASG; sampled bar thicknesses 0.2, 0.4, 0.6, 0.8 and 1.0 mm; height 10 mm; other detector geometry PT = 1.0 mm, REF = 0.3 mm, MgO and 0.10 mm Pb interlayer
ASG condition: 0.60 mm bars, 10 mm height	Monoenergetic 80 keV photons; normally incident 16 × 16 mm^2 planar source; source z = −150 mm; 100 mm HDPE scatterer; 5 mm gap; 20,000 primary photons
ASG-height scan (Fig. 9)	W ASG, 0.60 mm bars; heights 0.2–5.0 mm; 80 keV photons; 16 × 16 mm^2 planar source; z = −150 mm; 100 mm HDPE scatterer; 5 mm gap; 20,000 primaries per height
ASG scoring quantities	Tot: total number of positive-energy-deposition Screen-step records; Neiabs: Screen-step records in CopyNumber 7 and 9; $Nei_{frac} = Nei_{abs}$/Tot
Simulation parameter	Value or setting

Table S3. Event-level statistical analysis of the neighboring-channel Screen-step fraction for the W ASG with 0.60 mm bar thickness and 10 mm height.

Metric	Verified value
Source and input	80 keV; 16 × 16 mm^2 planar source; 100 mm HDPE; 5 mm gap; 20,000 primaries
Recorded Screen steps, Tot	6,071,825
Neighboring Screen steps, Neiabs (channels 7 + 9)	712,589
Neighboring step fraction, Neiabs/Tot	0.117359937 (11.735994%)
Primaries with ≥1 Screen step	7,132/20,000 (35.660%)
Primaries with ≥1 step in channels 7 or 9	826/20,000 (4.130%)
Bootstrap resampling	1,000 bootstrap samples of 20,000 primary histories each; zero-step histories retained
SE of neighboring step fraction	0.004113218 (0.411 percentage points)
95% percentile interval for fraction	[0.109226611, 0.125460636] (10.923%–12.546%)
95% interval: any Screen step per primary	[0.349850, 0.363650]
95% interval: any neighboring step per primary	[0.038400, 0.044150]

Note: Bootstrap resampling used primary-photon histories, including histories with no Screen records, with resampling seed 20261014. The standard error and interval refer to the configuration in the table title.

Table S4. Primary-history bootstrap uncertainty in the phase-integrated optical response for the configurations in Fig. 10.

Metric	Estimate	Bootstrap SE	95% percentile interval
Baseline CTKint (%)	13.658	0.618	12.386 to 14.780
Optimized CTKint (%)	1.529	0.190	1.174 to 1.938

CTKint difference (percentage points)	−12.129	0.639	−13.273 to −10.804
CTKint relative change (%)	−88.805	1.480	−91.499 to −85.519
Baseline optical count ($\times 10^7$)	4.083354	0.051094	3.983764 to 4.181436
Optimized optical count ($\times 10^7$)	2.155476	0.019672	2.116157 to 2.192255
Optical-count relative change (%)	−47.213	0.827	−48.812 to −45.541

Note: Differences are optimized minus baseline. Relative changes are (optimized/baseline − 1) × 100%. For CTKint reported in percent, SE is in percentage points. Each of 2,000 resamples contained 500 EventID indices per configuration, synchronized across its 61 scan positions. The configurations were resampled independently. With indices synchronized across configurations as a sensitivity analysis, the CTKint-difference interval was −13.075 to −10.938 percentage points, and the optical-count relative-change interval was −48.547% to −45.852%. Both analyses gave the same direction of change.

Table S5. Slanted-edge fit-stability and cumulative-history diagnostics for the configurations in Fig. 11.

A. Bootstrap fit-stability diagnostics.

Configuration and metric	Estimate	Bootstrap SD	2.5th–97.5th percentile range
Baseline W10–90 (mm)	2.951	7.902	0.238 to 31.119
Optimized W10–90 (mm)	1.876	12.080	0.288 to 35.659
W10–90 difference (mm)	−1.075	14.553	−27.979 to 32.531
Baseline MTF50 (cycles mm^{-1})	0.163	0.478	0.015 to 2.019
Optimized MTF50 (cycles mm^{-1})	0.256	0.447	0.013 to 1.667
MTF50 difference (cycles mm^{-1})	0.093	0.647	−1.815 to 1.428

Note: The same fitted width determines W10–90 and MTF50. All 2,000 fits per configuration returned finite positive widths; 43 baseline fits and 35 optimized fits produced covariance warnings. These fits and all extreme widths were retained. The percentile ranges describe instability of the resampled fit and are not validated confidence intervals for detector performance. Both difference ranges include zero. Cross-configuration synchronized resampling also gave an MTF50-difference range spanning zero (−1.569 to 1.338 cycles mm^{-1}).

B. Cumulative-history diagnostic.

Histories per row	Baseline W10–90 (mm)	Optimized W10–90 (mm)	Baseline MTF50 (cycles mm^{-1})	Optimized MTF50 (cycles mm^{-1})
250	2.475	0.188	0.194	2.559
500	1.131	0.377	0.425	1.273
750	3.730	1.867	0.129	0.257
1,000	2.951	1.876	0.163	0.256

Note: Each estimate uses the central seven rows and the first N primary histories per row. The subsets are nested. The variation demonstrates sensitivity to the number of available histories and does not establish independent-seed convergence.
